# Derivation and validation of generalized sepsis-induced acute respiratory failure phenotypes among critically ill patients: a retrospective study

**DOI:** 10.1186/s13054-024-05061-4

**Published:** 2024-10-01

**Authors:** Tilendra Choudhary, Pulakesh Upadhyaya, Carolyn M. Davis, Philip Yang, Simon Tallowin, Felipe A. Lisboa, Seth A. Schobel, Craig M. Coopersmith, Eric A. Elster, Timothy G. Buchman, Christopher J. Dente, Rishikesan Kamaleswaran

**Affiliations:** 1 Department of Surgery, Duke University School of Medicine, Durham, NC 27707 USA; 2 Department of Surgery, Emory University School of Medicine, Atlanta, GA 30332 USA; 3 Division of Pulmonary, Allergy, Critical Care, and Sleep Medicine, Emory University, Atlanta, GA 30322 USA; 4 Surgical Critical Care Initiative (SC2i), Uniformed Services University of the Health Sciences, Bethesda, MD 20814 USA; 5 Department of Surgery, Uniformed Services University of the Health Sciences and Walter Reed National Military Medical Center, Bethesda, MD 20814 USA; 6 Henry M. Jackson Foundation for the Advancement of Military Medicine, Inc., Bethesda, MD 20817 USA; 7 Emory Critical Care Center and Department of Surgery, Emory University School of Medicine, Atlanta, GA USA; 8 Academic Department of Military Surgery and Trauma, Royal Centre for Defence Medicine, Birmingham, UK

**Keywords:** Acute respiratory failure, Critical care, Phenotype, Unsupervised machine learning, Sepsis-induced ARF

## Abstract

**Background:**

Septic patients who develop acute respiratory failure (ARF) requiring mechanical ventilation represent a heterogenous subgroup of critically ill patients with widely variable clinical characteristics. Identifying distinct phenotypes of these patients may reveal insights about the broader heterogeneity in the clinical course of sepsis, considering multi-organ dynamics. We aimed to derive novel phenotypes of sepsis-induced ARF using observational clinical data and investigate the generalizability of the derived phenotypes.

**Methods:**

We performed a multi-center retrospective study of ICU patients with sepsis who required mechanical ventilation for ≥ 24 h. Data from two different high-volume academic hospital centers were used, where all phenotypes were derived in MICU of Hospital-I (N = 3225). The derived phenotypes were validated in MICU of Hospital-II (N = 848), SICU of Hospital-I (N = 1112), and SICU of Hospital-II (N = 465). Clinical data from 24 h preceding intubation was used to derive distinct phenotypes using an explainable machine learning-based clustering model interpreted by clinical experts.

**Results:**

Four distinct ARF phenotypes were identified: A (severe multi-organ dysfunction (MOD) with a high likelihood of kidney injury and heart failure), B (severe hypoxemic respiratory failure [median P/F = 123]), C (mild hypoxia [median P/F = 240]), and D (severe MOD with a high likelihood of hepatic injury, coagulopathy, and lactic acidosis). Patients in each phenotype showed differences in clinical course and mortality rates despite similarities in demographics and admission co-morbidities. The phenotypes were reproduced in external validation utilizing the MICU of Hospital-II and SICUs from Hospital-I and -II. Kaplan–Meier analysis showed significant difference in 28-day mortality across the phenotypes (*p* < 0.01) and consistent across MICU and SICU of both Hospital-I and -II. The phenotypes demonstrated differences in treatment effects associated with high positive end-expiratory pressure (PEEP) strategy.

**Conclusion:**

The phenotypes demonstrated unique patterns of organ injury and differences in clinical outcomes, which may help inform future research and clinical trial design for tailored management strategies.

**Supplementary Information:**

The online version contains supplementary material available at 10.1186/s13054-024-05061-4.

## Introduction

Sepsis is a heterogeneous syndrome that is characterized by life-threatening organ dysfunction due to a dysregulated host response to infection [1]. Despite advances in our knowledge and improvements in management strategies, sepsis continues to be one of the leading causes of death worldwide and remains a serious medical emergency [2, 3]. Patients with sepsis who develop acute respiratory failure (ARF) requiring mechanical ventilation represent a unique and complex subgroup [4–6]. ARF is one of the most common complications in sepsis and one of the strongest risk factors for mortality. This subgroup of patients also has heterogeneous risk factors, etiologies, pathophysiology, and immunopathogenic responses that contribute to ARF, as well as divergent clinical courses and outcomes [7]. A typical manifestation of ARF in patients with sepsis is the acute respiratory distress syndrome (ARDS) [8]. In addition to ARDS, these patients also frequently develop other extra-pulmonary organ dysfunctions that lead to increased complications and high mortality.

Clinically recognizable phenomena that are widely observed in sepsis and ARF, such as vital sign abnormalities (dyspnea, hypotension, tachypnea, oxygen desaturation, etc.) and laboratory abnormalities (lactic acidosis, hypoxemia and/or hypercapnia on arterial blood gas, etc.), are only superficial representations of complex pathophysiological and environmental interactions. Furthermore, they do not provide specific information regarding the heterogeneous clinical trajectories and outcomes, which contribute to the ongoing challenges in developing targeted management strategies and improving outcomes in sepsis-induced ARF. However, there may be subtle patterns of physiologic data and clinical features that may be unclear to clinicians at the bedside but are uncovered with the aid of machine learning (ML) models. The ML models can help recognize these patterns as unique phenotypes within heterogeneous syndromes such as sepsis-induced ARF. While latent class analysis (LCA) techniques have been used to derive hyper- and hypo-inflammatory phenotypes in ARDS with potential differences in treatment responses, these phenotypes only apply to a specific subset of patients with a key manifestation of ARF [9–11]. In addition to ARDS, there is a need to investigate other organ dysfunctions that are related to sepsis-induced ARF. Moreover, there is a need to identify generalizable phenotypes among patients with sepsis to elucidate possible mechanisms of complex clinical courses and targetable features pertaining to certain phenotypes.

We sought to utilize pre-intubation clinical data to develop generalizable phenotypes to investigate more complex multi-organ failure trajectories observed within this cohort [8]. We further sought to compare the derived generalized sepsis-induced ARF phenotypes against the binarized hyper- and hypo-inflammatory subphenotypes to characterize potential overlaps between these approaches. To understand the latent phenotypes in critically ill patients with sepsis-induced ARF, we developed a multi-phased unsupervised ML model to systematically identify novel phenotypes using multi-variable data collected from electronic medical records (EMR). Since the phenotyping involved multiple stages or phases, like imputation, feature scaling, feature selection, feature reduction, and k-means clustering, we termed it as “multi-phased” model.

## Methods

### Study design

This is a multi-center retrospective cohort study conducted at two high volume academic hospitals located in the southeastern United States (Atlanta, GA). Adult patients (≥ 18 years) admitted to the medical or surgical intensive care units (MICU or SICU) at either of these two metropolitan academic hospitals, Emory University Hospital (Hospital-I) and Grady Memorial Hospital (Hospital-II) with sepsis (based on the sepsis-3 criteria [1]) between 2016 and 2021 and developed ARF during their hospital admission were included [12]. Emory is a quaternary care hospital specializing in the care of adult critically ill patients, whereas Grady is known as a safety-net hospital. ARF was defined as requiring ≥ 24 h of invasive mechanical ventilation (IMV) for medical ICU patients. For surgical ICU patients, it is defined as ≥ 24 h of IMV even after 48 h from surgery.

### Participants

IMV patients included in our cohort were adult patients admitted to the hospital who were diagnosed with sepsis and during their hospital course required mechanical ventilation as well as adult surgical patients whose post-operative course was complicated, requiring *post-surgical IMV* lasting at least 24 h. Here, *post-surgical IMV* refers to initial IMV, re-ventilation or remaining in IMV state after 48th hour from the surgery completion. Due to our interests in identifying the phenotypes early in the course of sepsis-induced ARF, we utilized up to 24 h of data preceding the time of index IMV (or post-surgical IMV) in the study. Data collected from the EMR including laboratory values and vital signs, were used for phenotyping. A complete list of these clinical factors or features can be found in Supplementary file 1: Table E1 in the online data supplement. All factors represent clinical values that were routinely collected and recorded in the EMR. A set of demographic variables (e.g., age, sex, race, ethnicity), mortality, and comorbidity information were also included for further analysis of derived phenotypes. We created two separate datasets corresponding to MICU and SICU patients. We defined index time of IMV in the MICU dataset as the time at which the first mechanical ventilation parameters [positive end-expiratory pressure (PEEP), tidal volume, and/or plateau pressure] were recorded in the EMR in patients who met the above inclusion criteria; while for SICU dataset, it is the time of first ventilation parameters recorded from 48-h mark after the surgery.

**Fig. 1:**
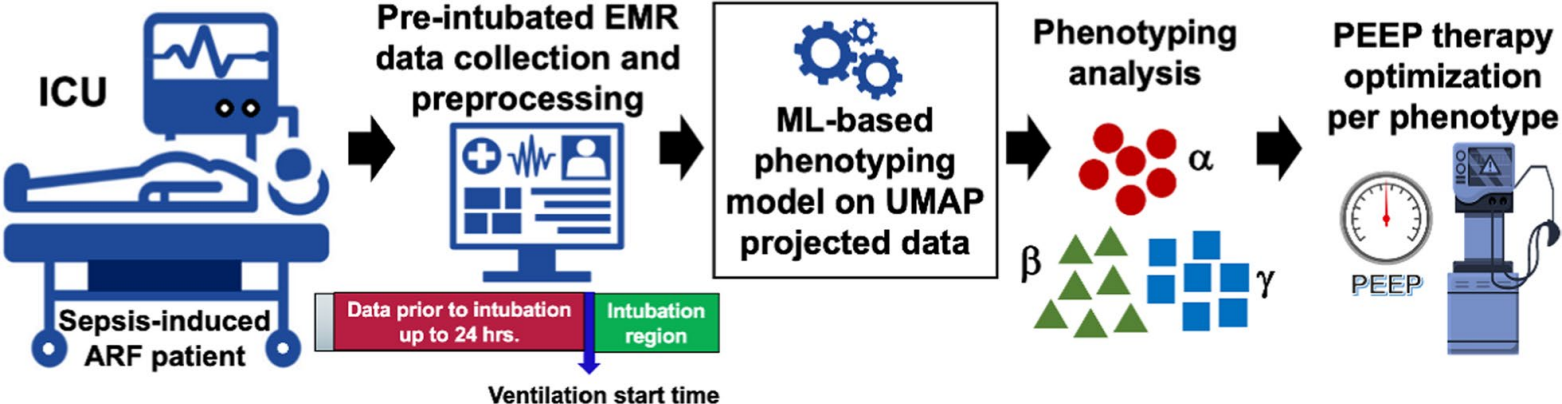
Overall study pipeline of our ARF phenotyping approach showing data extraction, preprocessing, feature reduction, model development for clustering, phenotype analysis and potential usage

We excluded patients who did not meet sepsis-3 criteria, patients admitted to neurological ICUs, patients admitted to the ICU post-operatively who were ventilated only for ≤ 24 h after 48 h from their surgeries, or those whose EMR data did not include any hourly collected physiological data up to 24 h prior to IMV. To derive enriched phenotypes for sepsis-induced ARF, we developed a high-fidelity unsupervised ML-based approach that incorporates a broad set of routinely collected clinical variables. The overall study pipeline is shown in Fig. 1.

### Procedure

We adopted a multi-center derivation and validation study design by first deriving phenotypes using medical ICU (MICU) data from Emory University Hospital, and then validating this phenotyping algorithm against MICU data collected from Grady Memorial Hospital. The phenotyping was further validated with surgical ICU (SICU) data from both the hospitals. The two hospitals serve unique and diverse patient populations located within the metropolitan southeast United States. Data used to derive and validate our algorithm were collected from the same years across the two hospitals.

In our study, we used EMR variables to implement sepsis-3 criteria for the identification of sepsis patients [1]. The implementation involves four major steps: (a) calculation of six individual Sequential Organ Failure Assessment (SOFA) scores for different organ systems, (b) suspicion time estimation based on administration of antibiotics (oral or parenteral) and blood cultures, (c) estimation of acute SOFA increase by two, and (d) sepsis-3 time estimation within a specified period around the suspicion time. By following this approach, sepsis patients were identified [1].

We applied the median aggregation across all routinely collected clinical features over the 24-h pre-ventilation period for each patient in our cohort. The motivation for using all readily available clinical features was to enable the model to achieve data-driven separability based on multi-organ dynamics. We dropped features that were missing in > 85% of patients in the aggregated data. To handle the outliers, they were treated as missing entries. Subsequently, we used multivariate imputation by chained equations (MICE) algorithm on the training data [13] to impute missing data.

Finally, we performed a Pearson’s correlation analysis, where a coefficient threshold of 0.75 was used to drop certain highly correlated features, resulting in 50 clinical features (listed in the Supplementary file 1: Table E1). We then normalized the data and used Uniform Manifold Approximation and Projection (UMAP) method to reduce dimensionality of the multivariate dataset and project onto a two-dimensions [14]. The optimal number of clusters and transformed feature dimension were decided by achieving a combination of highest silhouette score, highest Calinksi-Harabasz score and lowest Davies–Bouldin score for the clustering. UMAP and principal component analysis (PCA) transformations were explored in feature dimensionality reduction for various dimensions and clusters. Additionally, reconstruction error between the reconstructed data from transformation-embedding and the original data was also evaluated. Finally, we used a *k*-means (centroid-based) clustering algorithm that yielded four clusters. The derived clusters were analyzed for their most important features using SHapley Additive exPlanations (SHAP) values [15]. The derived phenotypes were then examined and interpreted by physicians P.Y., C.M.D. and C.M.C.

#### Validation of the phenotypes in external dataset

We trained a multivariate and multiclass logistic regression (LR) ML model for phenotype prediction on the derivation data with high accuracy. We used a scaled feature-set of 50 selected variables, from the derivation data, as input to the model. The data was randomly split into training (80%) and testing (20%) sets, respectively. The LR model outperformed other ML models such as random forests, support vector machines (SVM) and Gaussian Naïve Bayes classifier on the test-set. As per the TRIPOD guidelines, more details can be found in Supplementary file 1: Tables E4 and E5, and Figures E5 and E6 in the online data supplement. Finally, we applied this trained classifier model to the validation datasets. The purpose was to evaluate the robustness of the classifier in identifying similar phenotypes in ‘unseen’ and external data from a different medical center.

#### Estimation of treatment effects of high PEEP within phenotypes

We performed an exploratory analysis to examine whether the phenotypes would demonstrate different outcomes or clinical patterns in relation to high PEEP (PEEP ≥ 10) treatment. We conducted an analysis to estimate the effects of high PEEP (PEEP ≥ 10) on the derivation set using a propensity score matching (PSM) scheme on 28-day short-term mortality, by considering lab-values, vitals, demographics, and clinical scorings as confounding variables (see Supplementary file 1: Table E13). More details of our analysis are available in the Supplementary file 1: Table E14. We estimated the average treatment effects (ATE) along with the effect size. After matching, we also plotted Kaplan–Meier curves between patients who received high PEEP and those who did not within each of the phenotypes.

To further investigate how the contributed phenotypes compare to the existing work in the field, we sought to compare the proposed sepsis-induced ARF phenotypes to the ARDS hyper- and hypo-inflammatory subtypes [9–11, 16]. More details can be found in Supplementary file 1: Appendix-1 (A1.1) of the Online Data Supplement.

#### Analysis on COVID-19

We evaluated the distribution of data collected during COVID-19 years (2020–2021) and pre-COVID-19 years. We also analyzed the diagnosis codes to find COVID-19 patients within our study cohort and their distribution across phenotypes.

### Statistical analysis

Statistical analyses were performed using python libraries. Patient characteristics and endotype factors that represent continuous variables were analyzed using a Kruskal–Wallis test. Categorical variables were analyzed using a Chi-squared test. A multivariate log rank test was performed when comparing multiple variables and a *p* value of ≤ 0.05 was used for statistical significance.

## Results

### Patient characteristics

In this retrospective study, a total of 3349 encounters from 3225 unique patients admitted to MICU at Emory University Hospital (Atlanta, GA) were selected from the derivation data for initial phenotyping. The cohort in our study consists of patients across a wide range of demographic variables, such as age (mean: 62.3 ± 15.3 years), sex (male: 53.1%), and race (Caucasian: 42.2%, African American: 47.4%). For validating our phenotyping algorithm, 867 encounters from N = 848 unique and diverse patients were selected from the MICU of Grady Memorial Hospital (Atlanta, GA). Characteristics of these cohorts are described in Tables 1 and 2. For validation of multi-ICU generalization, we used SICU patients from Emory [1128 encounters (N = 1112)] and Grady [466 encounters (N = 465)] hospitals, who required intubation even after 48 h of completion of their surgeries. Characteristics of these SICU patients are available in the Supplementary file 1: Tables E6 and E7.

**Table 1:** Summary of patient characteristics of the derivation cohort (Emory MICU) and its phenotypes

Parameters	Whole cohort	A	B	C	D	*p* value
Count (%)	3349 (100)	845 (25.2)	692 (20.7)	993 (29.7)	819 (24.4)	–
Mortality*	1295, 40.2%	337, 40.9%	353, 51.2%	205, 21.4%	400, 49.6%	–
Age, mean (std)	62.3 (15.5)	64.8 (14.6)	62.0 (15.0)	61.7 (16.7)	60.8 (15.0)	< 0.001
Males, count (%)	1814 (54.2)	492 (58.2)	363 (52.5)	547 (55.1)	412 (50.3)	–
Race: African American or Black, count (%)	1624 (48.5)	457 (54.1)	337 (48.7)	469 (47.2)	361 (44.1)	< 0.001
Race: Caucasian or White, count (%)	1442 (43.1)	341 (40.4)	280 (40.5)	453 (45.6)	368 (44.9)	
Ethnicity: Hispanic, count (%)	129 (3.9)	23 (2.7)	38 (5.5)	30 (3.0)	38 (4.6)	< 0.001
Ethnicity: Non-Hispanic, count (%)	2975 (88.8)	770 (91.1)	589 (85.1)	903 (90.9)	713 (87.1)	
P/F ratio, m (IQR)	240.0 [162.0, 334.9]	302.3 [226.7, 406.4]	123.3 [90.0, 185.0]	240.0 [185.0, 317.7]	266.5 [196.5, 346.7]	< 0.001
S/F ratio, m (IQR)	245.0 [188.5, 315.6]	250.0 [240.0, 326.7]	120.8 [97.5, 154.8]	248.8 [232.5, 320.4]	247.5 [220.0, 325.0]	< 0.001
FiO_2_, m (IQR)	0.4 [0.3, 0.5]	0.4 [0.3, 0.4]	0.8 [0.6, 1.0]	0.4 [0.3, 0.4]	0.4 [0.3, 0.4]	< 0.001
PaO_2_, m (IQR)	94.0 [77.0, 124.0]	107.0 [86.0, 146.1]	81.0 [67.0, 102.0]	92.0 [77.7, 116.1]	99.2 [79.6, 125.9]	< 0.001
PaCO_2_, m (IQR)	37.0 [32.0, 43.0]	37.0 [32.0, 43.0]	38.5 [33.0, 45.0]	39.0 [34.0, 46.9]	33.0 [29.0, 38.0]	< 0.001
MAP, m (IQR)	82.0 [75.0, 90.2]	79.0 [73.0, 87.5]	85.0 [77.0, 94.0]	87.0 [80.0, 95.0]	77.0 [72.0, 84.0]	< 0.001
Creatinine, m (IQR)	1.4 [0.9, 2.9]	3.5 [1.9, 5.7]	1.3 [0.8, 2.1]	1.1 [0.8, 1.5]	1.4 [0.9, 2.5]	< 0.001
Bilirubin total, m (IQR)	0.7 [0.5, 1.4]	0.8 [0.5, 1.4]	0.6 [0.4, 1.0]	0.6 [0.4, 1.1]	1.2 [0.6, 4.1]	< 0.001
Albumin, m (IQR)	3.0 [2.6, 3.5]	2.9 [2.5, 3.3]	3.1 [2.7, 3.5]	3.4 [3.0, 3.8]	2.6 [2.2, 2.9]	< 0.001
Lactic acid, m (IQR)	1.6 [1.2, 2.6]	1.5 [1.2, 2.1]	1.6 [1.2, 2.3]	1.4 [1.1, 2.0]	2.3 [1.4, 4.7]	< 0.001
D-dimer, m (IQR)	2678.2 [1141.2, 9258.5]	3177.0 [1427.0, 6981.5]	1573.0 [979.0, 4622.0]	1686.5 [897.8, 5183.0]	9828.0 [3187.0, 27,545.0]	< 0.001
Platelets, m (IQR)	184.0 [110.2, 265.8]	175.0 [112.0, 245.0]	209.0 [143.0, 286.0]	213.0 [149.0, 291.0]	118.0 [53.0, 202.8]	< 0.001
Hemoglobin, m (IQR)	9.8 [8.2, 12.0]	8.8 [7.8, 10.3]	11.0 [9.0, 12.8]	11.5 [9.7, 13.4]	8.6 [7.7, 10.2]	< 0.001
BNP, m (IQR)	328.0 [100.0, 915.0]	750.5 [251.2, 1775.5]	142.8 [60.0, 441.8]	279.0 [82.0, 664.0]	396.0 [147.5, 966.0]	< 0.001
BUN, m (IQR)	30.0 [19.0, 50.0]	56.0 [34.0, 80.2]	28.0 [17.5, 46.0]	22.0 [14.0, 33.0]	28.0 [18.0, 42.0]	< 0.001
SOFA max total, m (IQR)	7.0 [4.0, 9.0]	8.0 [5.0, 10.0]	6.0 [4.0, 8.0]	5.0 [3.0, 7.0]	8.0 [6.0, 11.0]	< 0.001
GCS total score, m (IQR)	14.0 [12.0, 15.0]	14.0 [12.0, 15.0]	15.0 [11.2, 15.0]	14.0 [12.0, 15.0]	14.0 [11.0, 15.0]	< 0.001
PEEP, m (IQR)	6.0 [6.0, 10.0]	6.0 [6.0, 8.0]	8.0 [6.0, 11.0]	6.0 [6.0, 8.0]	6.0 [6.0, 8.0]	< 0.001

For clinical variables, this table lists the medians and interquartile ranges (IQR: Q1–Q3) for each phenotype as well as for the whole cohort. The *p* value is also provided for each variable to indicate the statistical significance of the differences among the phenotypes. For evaluating statistical significance, Kruskal–Wallis test was performed for continuous variables and Chi-squared test was used for categorical variables. *Mortality was computed with respect to patients (not encounters). Abbreviations used**—**count, total encounters; mean, average; std, standard deviation; m, median; IQR, interquartile range; PaO_2_, partial pressure of oxygen; SpO_2_, peripheral oxygen saturation level; FiO_2_, fraction of inspired oxygen; P/F, PaO_2_/FiO_2_ ratio; S/F, SpO_2_/FiO_2_ ratio; PaCO_2_, partial pressure of carbon dioxide in arterial blood; MAP, mean arterial blood pressure; Resp., respiration; BNP, B-type natriuretic peptide; BUN, blood urea nitrogen; SOFA, sequential organ failure assessment; GCS, Glasgow coma scale. Measurement units**—**P/F ratio, PaO_2_, PaCO_2_, and MAP: mmHg; S/F ratio and FiO_2_: unitless; creatinine and bilirubin total: mg/dL; albumin: g/L; lactic acid: mmol/L; D-dimer: ng/mL; platelets: × 10^3^/µL; hemoglobin: g/dL; BNP: pg/mL; BUN: mg/dL

**Table 2:** Summary of patient characteristics of the validation cohort (Grady MICU) and its phenotypes

Parameters	Whole cohort	A	B	C	D	*p* value
Count (%)	867 (100)	214 (24.7)	49 (5.7)	404 (46.6)	200 (23.1)	–
Mortality*	294, 34.67%	80, 38.28%	34, 69.39%	83, 20.75%	97, 48.5%	–
Age, mean (std)	59.6 (15.1)	61.6 (13.9)	64.2 (13.1)	59.0 (16.1)	57.7 (14.3)	0.007
Males, count (%)	531 (61.2)	128 (59.8)	26 (53.1)	257 (63.6)	120 (60.0)	–
Race: African American or Black, count (%)	670 (77.3)	171 (79.9)	35 (71.4)	307 (76.0)	157 (78.5)	0.273
Race: Caucasian or White, count (%)	126 (14.5)	22 (10.3)	9 (18.4)	69 (17.1)	26 (13.0)	
Ethnicity: Hispanic, count (%)	39 (4.5)	18 (8.4)	2 (4.1)	9 (2.2)	10 (5.0)	0.029
Ethnicity: Non-Hispanic, count (%)	818 (94.3)	194 (90.7)	46 (93.9)	389 (96.3)	189 (94.5)	
P/F ratio, m (IQR)	267.5 [197.2, 343.6]	300.6 [242.5, 387.5]	104.0 [88.0, 154.5]	256.7 [200.0, 340.0]	272.0 [185.9, 327.6]	< 0.001
S/F ratio, m (IQR)	245.0 [200.0, 250.0]	250.0 [242.5, 250.0]	100.0 [94.8, 106.6]	245.0 [227.5, 250.0]	245.0 [198.0, 250.0]	< 0.001
FiO_2_, m (IQR)	0.4 [0.4, 0.5]	0.4 [0.4, 0.4]	1.0 [0.9, 1.0]	0.4 [0.4, 0.4]	0.4 [0.4, 0.5]	< 0.001
PaO_2_, m (IQR)	110.0 [87.0, 141.0]	131.0 [99.0, 159.0]	91.0 [75.0, 108.0]	107.0 [85.0, 136.0]	111.5 [87.0, 138.0]	< 0.001
PaCO_2_, m (IQR)	36.0 [31.0, 41.0]	34.0 [30.0, 39.0]	35.0 [30.0, 45.0]	38.0 [33.5, 43.0]	33.0 [29.0, 37.0]	< 0.001
MAP, m (IQR)	86.0 [78.0, 96.0]	82.1 [75.0, 89.9]	83.0 [77.5, 92.0]	92.8 [85.0, 103.1]	79.0 [74.0, 86.0]	< 0.001
Creatinine, m (IQR)	1.4 [0.9, 2.8]	4.1 [1.7, 6.9]	1.6 [1.1, 3.1]	1.1 [0.8, 1.6]	1.4 [0.8, 2.4]	< 0.001
Bilirubin total, m (IQR)	0.7 [0.5, 1.4]	0.6 [0.4, 1.1]	0.9 [0.5, 2.5]	0.7 [0.5, 1.2]	1.2 [0.6, 3.7]	< 0.001
Albumin, m (IQR)	3.0 [2.5, 3.6]	2.9 [2.5, 3.3]	3.0 [2.6, 3.5]	3.5 [3.1, 4.0]	2.2 [1.9, 2.6]	< 0.001
Lactic acid, m (IQR)	2.3 [1.7, 3.7]	2.1 [1.6, 3.2]	2.6 [1.9, 4.3]	2.2 [1.7, 3.3]	3.0 [2.0, 5.0]	< 0.001
D-dimer, m (IQR)	5220.0 [2041.0, 15,974.0]	5631.0 [2370.5, 21,648.0]	4898.0 [2668.0, 10,731.5]	3953.0 [1551.0, 7646.0]	8468.0 [2529.4, 22,770.5]	< 0.001
Platelets, m (IQR)	188.0 [119.0, 260.5]	178.5 [110.8, 255.5]	147.0 [97.0, 235.0]	213.5 [150.8, 276.2]	154.0 [83.5, 232.5]	< 0.001
Hemoglobin, m (IQR)	10.9 [8.7, 12.9]	9.4 [7.9, 11.4]	11.0 [8.9, 12.7]	12.2 [10.9, 14.0]	9.0 [7.7, 10.4]	< 0.001
BNP, m (IQR)	269.0 [105.0, 873.5]	590.0 [247.0, 1501.0]	222.0 [77.0, 653.0]	213.0 [92.0, 736.0]	215.5 [103.8, 675.5]	< 0.001
BUN, m (IQR)	27.5 [16.0, 51.0]	62.0 [40.0, 92.0]	34.0 [20.0, 52.0]	19.5 [13.0, 31.6]	23.0 [15.0, 41.0]	< 0.001
SOFA max total, m (IQR)	6.0 [4.0, 9.0]	8.0 [6.0, 10.0]	7.0 [5.0, 10.0]	5.0 [3.0, 7.0]	8.0 [5.0, 10.0]	< 0.001
GCS total score, m (IQR)	14.0 [11.0, 15.0]	14.0 [10.0, 15.0]	14.0 [12.0, 15.0]	14.0 [10.0, 15.0]	14.2 [12.0, 15.0]	0.117
PEEP, m (IQR)	8.0 [5.0, 8.0]	8.0 [5.0, 8.0]	10.0 [8.0, 10.0]	8.0 [5.0, 8.0]	8.0 [5.0, 8.0]	< 0.001

For clinical variables, this table lists the medians and interquartile ranges (IQR: Q1–Q3) for each phenotype as well as for the whole cohort. The p-value is also provided for each variable to indicate the statistical significance of the differences among the phenotypes. For evaluating statistical significance, Kruskal–Wallis test was performed for continuous variables and Chi-squared test was used for categorical variables. *Mortality was computed with respect to patients (not encounters). Abbreviations used**—**count, total encounters; mean, average; std, standard deviation; m, median; IQR, interquartile range; PaO_2_, partial pressure of oxygen; SpO_2_, peripheral oxygen saturation level; FiO_2_, fraction of inspired oxygen; P/F, PaO_2_/FiO_2_ ratio; S/F, SpO_2_/FiO_2_ ratio; PaCO_2_, partial pressure of carbon dioxide in arterial blood; MAP, mean arterial blood pressure; Resp., respiration; BNP, B-type natriuretic peptide; BUN, blood urea nitrogen; SOFA, sequential organ failure assessment; GCS, Glasgow coma scale. Measurement units**—**P/F ratio, PaO_2_, PaCO_2_, and MAP: mmHg; S/F ratio and FiO_2_: unitless; creatinine and bilirubin total: mg/dL; albumin: g/L; lactic acid: mmol/L; D-dimer: ng/mL; platelets: × 10^3^/µL; hemoglobin: g/dL; BNP: pg/mL; BUN: mg/dL

### Phenotyping results

**Fig. 2:**
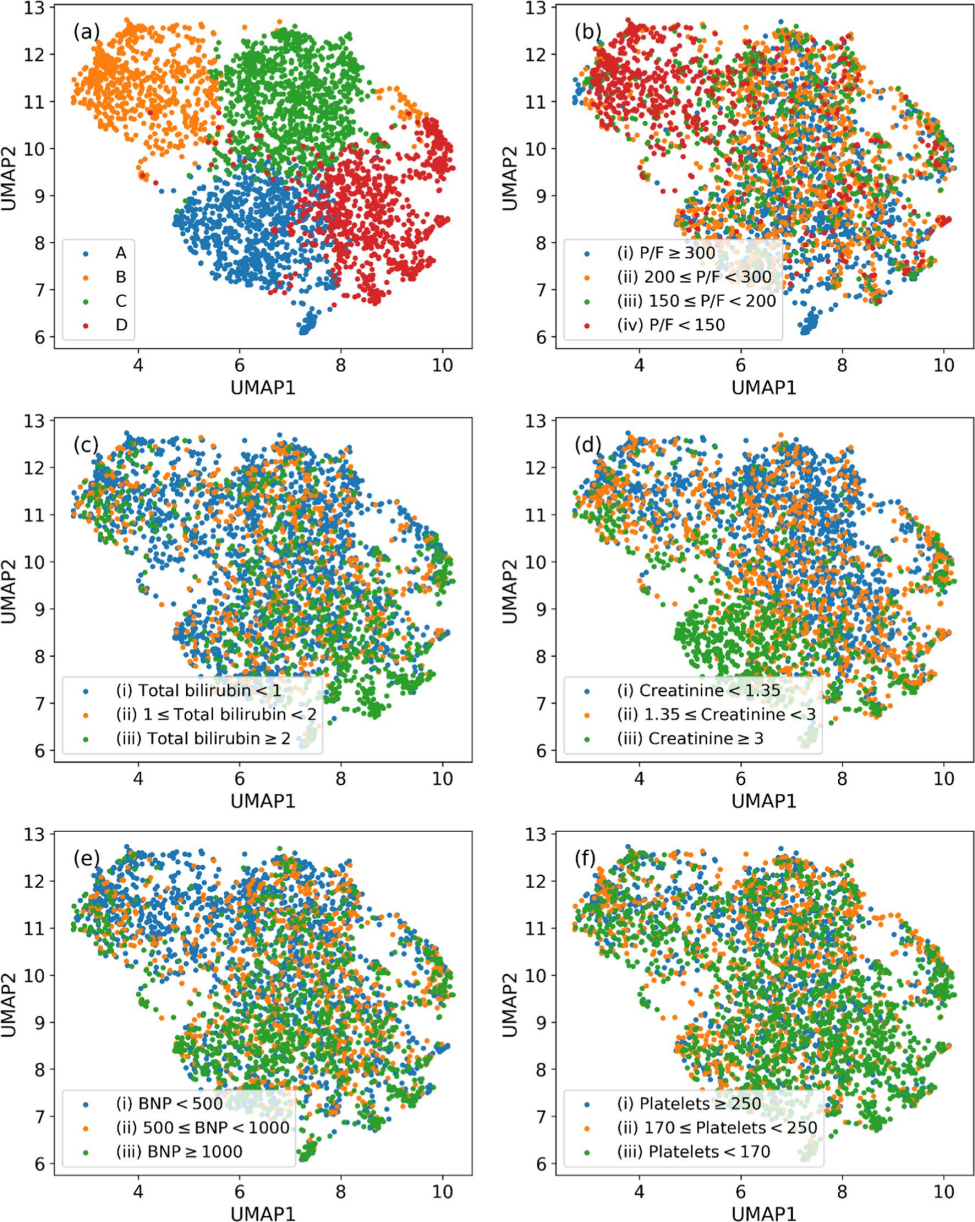
UMAP projections showing distribution of the derivation data for ARF phenotyping and feature variations. **a** UMAP representing all ARF phenotypes, and **b**–**f** UMAP representations showing variabilities in P/F ratio (mmHg), bilirubin total (mg/dL), creatinine (mg/dL), BNP (pg/mL), and platelets (× 10^3^/µL), respectively

Our clustering algorithm for deriving enriched ARF phenotypes characterized by various risks profiles yielded four clusters on the derivation data with a silhouette score of 0.418, Calinksi–Harabasz score (variance ratio criterion) of 3516.74, and Davies–Bouldin score of 0.79. More insights on selecting optimal number of clusters and UMAP dimension are provided in the Supplementary file 1: Table E2. Also, a bar graph is shown in Supplementary file 1: Figure E1 illustrating mean square reconstruction error (MSE) for various UMAP dimensions. The obtained MSEs are 0.2959, 0.2850, 0.2735 and 0.2683 for dimensions 2, 3, 4 and 5, respectively. We observed a quite small change in MSE (~ 0.01), further supporting our selection of UMAP dimension of 2. The patient distributions are shown in Fig. 2 using a 2-D UMAP representing formed clusters and variations of important clinical features across these distributions. SHAP values were used to identify the important features that distinguished one cluster from another. SHAP plots are available in Supplementary file 1: Figure E4 in the online data supplement. For more details on SHAP, please refer to Supplementary file 1: Appendix-2 in the online data supplement. Subsequently, critical care physician experts helped interpret and characterize the four clusters as phenotypes based on their characteristics.

Table 1 summarizes the clinical and demographic variables for each of the four derived ARF phenotypes along with their mortality outcomes. The first phenotype (N = 825 patients) has ARF patients with multiple laboratory abnormalities, such as highest median levels of creatinine (median: 3.47, IQR: 1.89–5.74 mg/dL), blood urea nitrogen (BUN) (median: 56, IQR: 34–80.25 mg/dL) and B-type natriuretic peptide (BNP) (median: 750.5, IQR: 251.25–1775.5 pg/mL). Based on the characteristics, we named this phenotype A (MOD-1) with *severe multiple organ dysfunction (MOD) showing a high likelihood of kidney injury and heart failure*. The second phenotype (N = 689 patients) consists of patients with severe hypoxia and clinical characteristics suggestive of non-radiographic features of severe ARDS (low partial pressure of oxygen (PaO_2_) to fraction of inspired oxygen (FiO_2_) ratio [P/F ratio] [median: 123, IQR: 90–185 mmHg] and high FiO_2_ [mean: 0.8, IQR: 0.6–1]) and has the highest mortality (51%). We called it phenotype B (*severe hypoxemic respiratory failure*). The third phenotype (N = 959 patients) consists of patients with no evidence of organ failure other than mild hypoxia (median P/F ratio: 240 [IQR: 185–317.7]) and normal lactic acid levels (median: 1.42 mmol/L). We called it phenotype C (*mild hypoxia*)**.** The fourth phenotype (N = 806) consists of ARF patients with highest total bilirubin (median: 1.2, IQR: 0.6–4.1 mg/dL) and highest D-dimer levels, lowest platelets (median: 118, IQR: 53–202.8 × 10^3^/µL) and highest lactic acid (median: 2.35, IQR: 1.43–4.67 mmol/L) suggesting multi-system organ dysfunction. As such, we named this phenotype D (MOD-2) with *severe MOD showing a high likelihood of hepatic injury, coagulopathy and lactic acidosis***.** From Table 1, we observed that phenotype B has the highest mortality (51%), followed by phenotype D (49.6%) and phenotype A (40.9%). The relatively healthier phenotype B had a mortality of 21.4%. Phenotype D is also characterized by the highest proportion of patients with septic shock (n = 651 patient encounters (79.5%), whereas C consists of the lowest proportion of septic-shock patients (450 encounters, 45.3%). Thus, our phenotypes not only identified distinct patterns of organ injury in patients with sepsis-induced ARF, but also different rates of mortality and septic-shock distributions. We also used diagnosis codes to highlight the most frequently occurred diseases per phenotype for further validating our characterization and naming of phenotypes. They are listed in the Supplementary file 1: Table E3. To confirm more insights of MOD profiles in phenotypes, a set of all six individual SOFA were analyzed from the pre-intubation window for each phenotype. The maximum method was used for their aggregation. Supplementary file 1: Table E9 presents SOFA score-maps for Emory MICU data, where the findings clearly align with our phenotype characterizations.

**Fig. 3:**
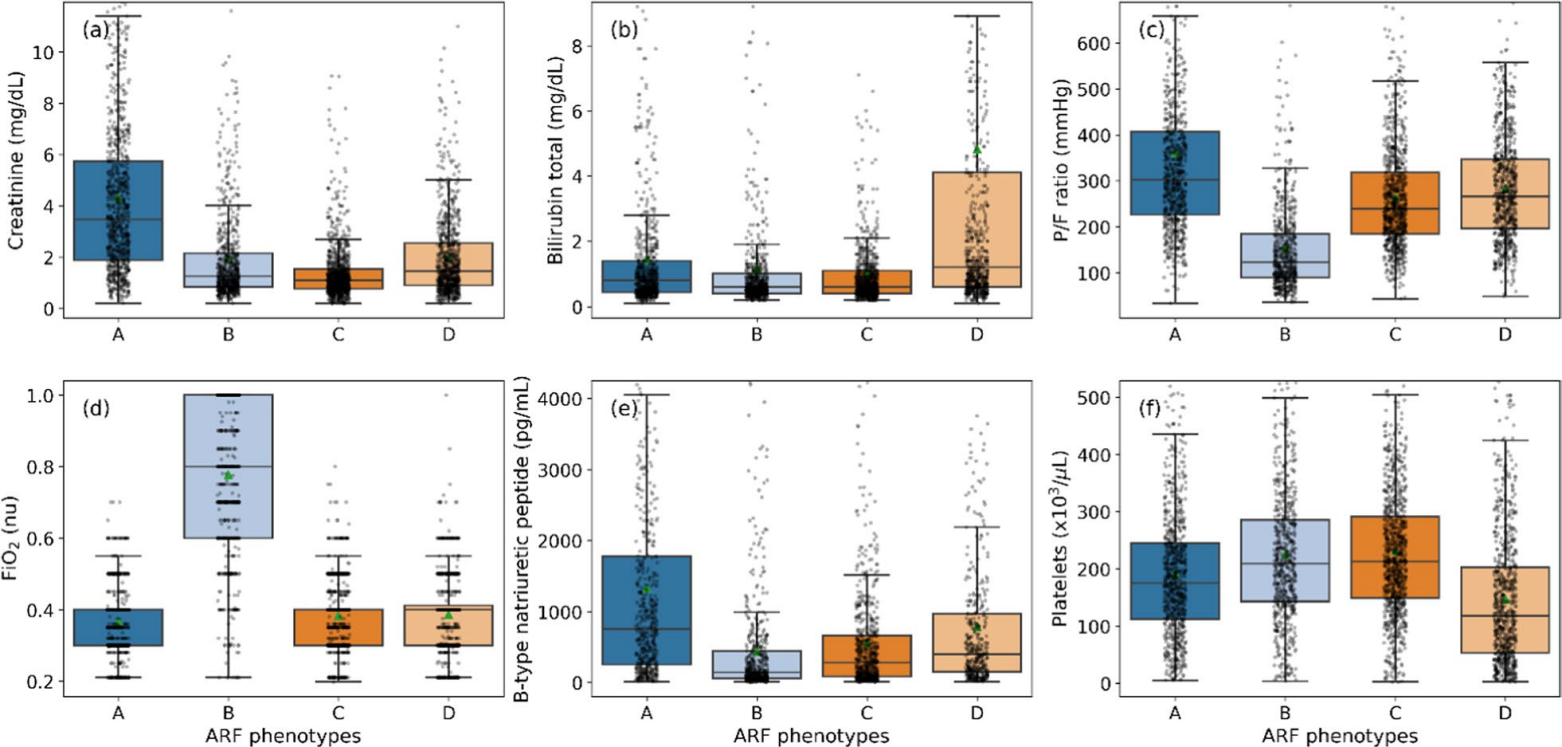
Visualization of feature variations across different ARF phenotypes of the derivation data. **a**–**f** boxplot representations of different phenotypes showing variabilities in creatinine (mg/dL), bilirubin total (mg/dL), P/F ratio (mmHg), FiO_2_, BNP (pg/mL), and platelets (× 10^3^/µL), respectively to show fitness of individual organs. Presented phenotyping results were significant (*p* < 0.001, Kruskal–Wallis test) for each of the variables

Boxplots were drawn to illustrate the variabilities in certain prominent features such as creatinine (renal), total bilirubin (hepatic), P/F ratio and FiO_2_ (respiratory), BNP (cardiac), and platelets (coagulopathy), as shown in Fig. 3a–f. We also calculated the age-adjusted Charlson Comorbidity Index based on admission diagnosis ICD-9 codes for all four phenotypes, and they are 2.3 (95% CI 2.21, 2.4), 1.94 (95% CI 1.84, 2.05), 1.99 (95% CI 1.9, 2.07) and 2.06 (95% CI 1.97, 2.16), respectively.

### External and multi-specialty validation of sepsis-induced ARF phenotypes

To validate our phenotyping algorithm, we utilized an external hospital’s MICU cohort from Grady Memorial Hospital, Atlanta, GA. Our methodology involves training a supervised learning (logistic regression) classifier on the derivation dataset to predict the corresponding phenotype. Thereafter, we employed the trained model on the validation dataset to determine the phenotype for each patient encounter. We summarize the phenotype validation results in Table 2. These results indicate that most features across the four phenotypes remain consistent in the validation dataset, highlighting the reliability and generalizability of our phenotyping approach.

For further analysis of the phenotypes, Supplementary file 1: Figure E2 in the online data supplement shows radar diagrams illustrating average variations of all clinical feature values across four formed phenotypes of the derivation and validation data, where all features are normalized in the range 0–1. Additionally, radar diagrams in Supplementary file 1: Figure E3 in the online data supplement presents distributions of demographic variables and mortality outcomes across various phenotypes of the derivation and validation data. Additionally, our phenotyping approach was also validated on SICU cohorts of both Emory and Grady hospitals. Their phenotyping results are listed in the Supplementary file 1: Tables E6 and E7. From the phenotyping results, we observed the following points: (a) all phenotypes were identified in validation cohorts; (b) the prevalence of the phenotypes had marked differences across datasets, with phenotype B prevalence being very low (< 6%) across all validation sets; and (c) some of the differences in phenotype prevalence may reflect differences in patient case mix across the ICUs. We also analyzed aggregated pre-intubated individual SOFA for these datasets, and the results are listed in Supplementary file 1: Tables E10–E12. They show consistency in earlier results obtained from the derivation data. Results on further characterization of the proposed phenotypes as hyper- or hypo-inflammatory can be found in Supplementary file 1: Appendix-1 (A1.2) of the online data supplement.

### Short-term survival analysis

**Fig. 4:**
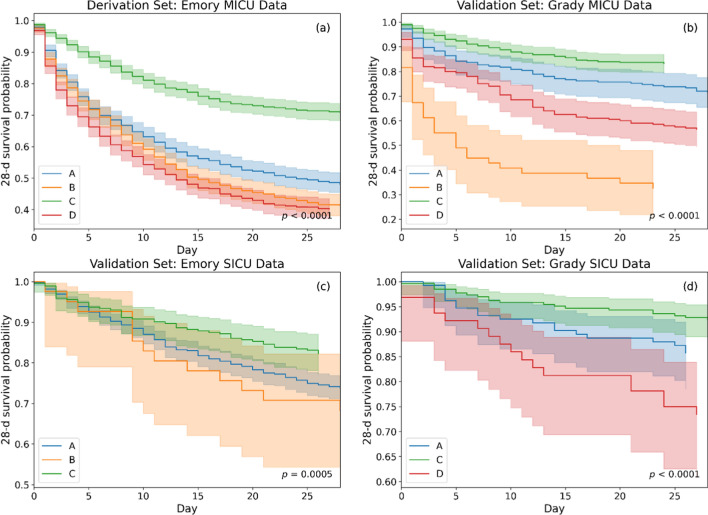
Kaplan–Meier curve showing 28-day survival rates for ARF patients stratified by phenotypes developed for **a** derivation set: Emory MICU data, and **b** validation set: Grady MICU data, **c** validation set: Emory SICU data, and **d** validation set: Grady SICU data. Survival was analyzed from the time of intubation. Survival probabilities (solid line) with their confidence intervals (faded region) are represented for different phenotypes via different color-codes

Trajectory of short-term outcomes can provide a better differentiation among phenotypes. For a 28-day short-term analysis, average vent-free days (VFD) were found as 10.4, 8.6, 15.4 and 8.5, respectively for phenotypes A to D of the derivation set. To evaluate the survival probability of patients in each phenotype, we plotted Kaplan–Meier curves [17] for a 28-day period following intubation, as shown in Fig. 4. The analysis was performed for derivation and validation datasets, where survival traces of phenotype D of Emory SICU (N = 12) and B of Grady SICU (N = 5) were omitted here due to having their small sample sizes. We observed that the mortality trends across various phenotypes were consistent for MICU and SICU of both centers (*p* value for trend < 0.001), with phenotype C having the best survival followed by A, and phenotypes B and D having the poor survival rates in both centers. This suggests that our phenotyping approach is generalizable in identifying the least and the most critical phenotypes in terms of short-term survival for ARF patients with different demographic characteristics.

### Exploratory analyses of clinical differences among the phenotypes

**Fig. 5:**
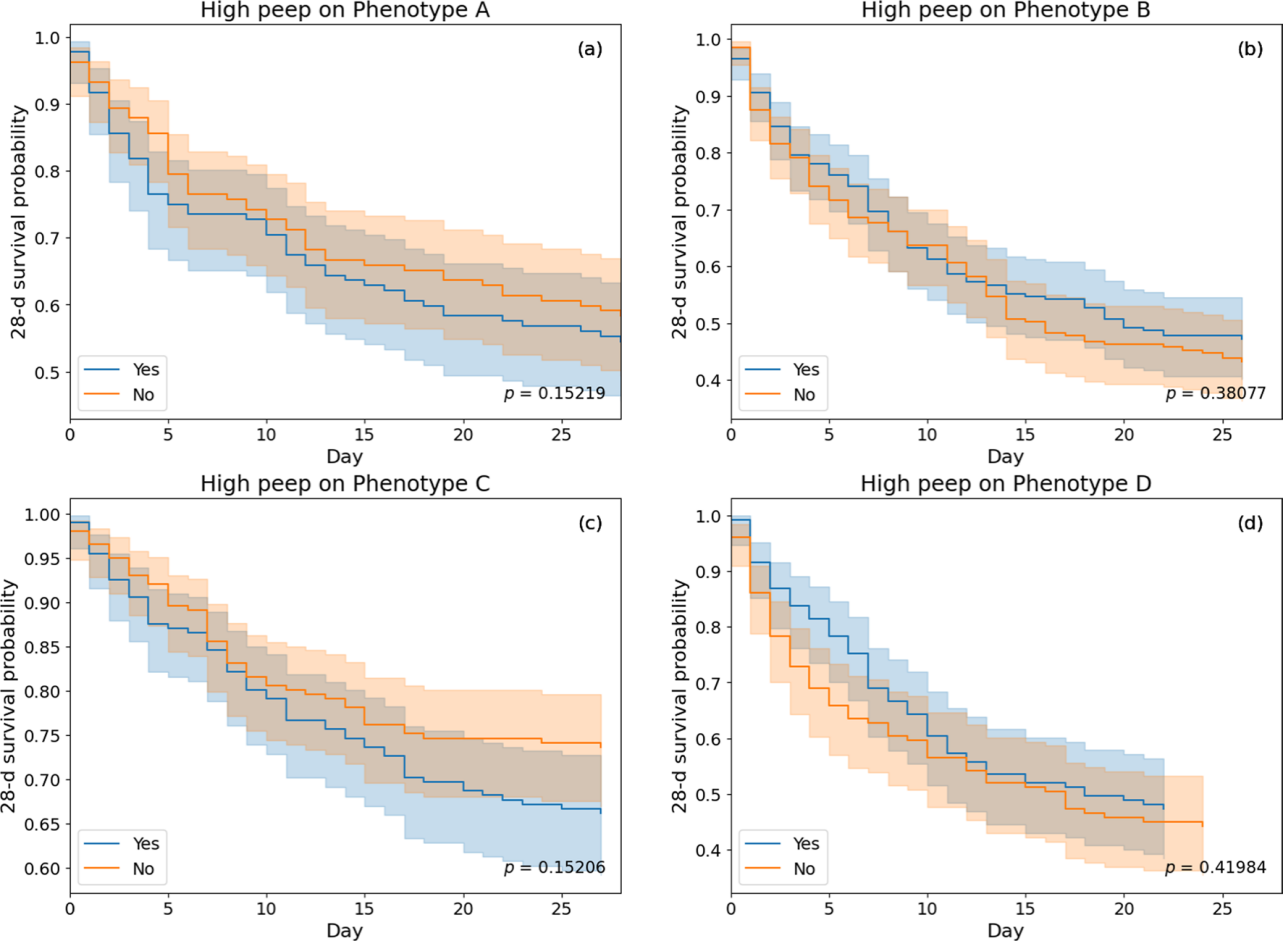
Kaplan–Meier survival curves showing treatment effects of high PEEP (PEEP ≥ 10) and low PEEP (< 10) regimes on propensity matched ARF phenotypes from the derivation dataset. Survival probabilities (solid line) with their 95% confidence intervals (faded region) are represented for treated and untreated cohorts within each phenotype

Within phenotypes, 16.7% in A, 49.6% in B, 24% in C, and 16.2% in D were administered with PEEP ≥ 10 regime on mechanical ventilator. After propensity score matching, the matching sizes for the four phenotypes were found to be 132, 201, 201 and 129, respectively. The ATE with 95% confidence intervals were obtained as 0.04 (− 0.08, 0.16) for A, − 0.04 (− 0.14, 0.06) for B, 0.07 (− 0.02, 0.17) for C, and − 0.03 (− 0.15, 0.08) for D. A negative ATE suggests reduced mortality outcomes for the treated group. We also plotted the effect sizes (standardized mean differences) of variables for each phenotype before and after matching (see Supplementary file 1: Figures E8–E11). Kaplan–Meier curves were also plotted in Fig. 5 to show the effect of high PEEP within each of the phenotypes.

In phenotype B with severe hypoxic respiratory failure, higher PEEP (≥ 10) was associated with better survival (negative ATE) than lower PEEP (< 10), but the opposite association was seen in phenotype C. Among both MOD phenotypes, higher PEEP was found effective for D, whereas it was ineffective for A. We must emphasize that this analysis was purely exploratory in nature and was carried out to examine the feasibility of further research on the treatment effects of various therapies.

### Practice variance during COVID-19

When evaluating the derivation strategy independently during the 2020–2021 data, we found that they were consistent with that of pre-COVID-19 years, without significant variance in the distribution of the phenotypes. Relevant details on the sensitivity analyses are available in Supplementary file 1: Figure E7 in the online data supplement. With the ICD-10 diagnosis codes, we observed that out of 3349 patient encounters, 568 were diagnosed with COVID-19 on the derivation set. The year-wise distribution of COVID-19 patients was found to be 356 and 212 from years 2020 and 2021, respectively. A majority of these patients (289, 42% of 692) were under phenotype B, whereas 73 (9% of 845), 137 (14% of 993) and 69 (8% of 819) were found in phenotypes A, C and D, respectively. Details on validation sets can be found in the Supplementary file 1: Table E8. A major portion of the COVID-19 patients come under phenotype B in all ICUs, except in Grady SICU possibly due to the scarcity of data. Thus, COVID-19 could be related to the prevalence of Phenotype B.

## Discussion

In this study, we performed UMAP projection and unsupervised clustering to derive novel phenotypes of sepsis-induced ARF. The phenotypes derived using early clinical data from the pre-intubation phase of sepsis-induced ARF not only demonstrated unique patterns of organ injury, but also correlated with differences in mortality and exhibited potential differences in outcomes in relation to High vs. Low PEEP strategy. Furthermore, the characteristics of the phenotypes remained consistent in the validation datasets. The performance was evaluated on comprehensive datasets including rich and diverse cohorts of patients with varying comorbidities across different demographics.

Sepsis and ARF are both heterogeneous syndromes with diverse risk factors, etiologies, clinical presentations, prognosis, pathophysiology, and immune response mechanisms that continue to pose limitations for improving outcomes. The present study is unique in that it identified novel phenotypes in a broader population of patients with sepsis-induced ARF, and provides valuable information about their distinct clinical characteristics, outcomes, and potential differences in treatment responses. Prior studies have tried to address this issue by deriving phenotypes separately in sepsis and in ARDS, but have not focused on phenotypes of sepsis-induced ARF [11, 18]. For example, Aliberti et al*.* described four phenotypes of patients with community-acquired pneumonia (CAP) in presence of ARF or severe sepsis (SS): CAP without ARF or SS, CAP with ARF only, CAP with SS only and CAP with both ARF and SS [19]. Essay et al*.* presented an algorithm for phenotyping ARF patients using remotely monitored ICU (tele-ICU) data from more than 200 patients and validated it using a large cohort EMR data from 46 ICUs in southwest United States [20]. The validation was done by comparing the output of the phenotyping algorithm to a manual review, and the common causes of misclassification were noted [21]. Unlike our work, this study does not characterize various degrees of MOD associated with ARF and the severity of such outcomes. It only characterizes patients based on the sequence of different respiratory support they received. The set of features analyzed is also limited; in contrast, this work considers an expanded set of clinical features for phenotyping. While many of these studies identify phenotypes in sepsis at large [22–26], they have not examined phenotypes that may exist specifically within sepsis-induced ARF. Similarly, LCA-derived phenotypes of ARDS have been well-studied, but do not directly apply to ventilated patients with sepsis who do not satisfy the Berlin definition of ARDS.

Although previously studies have highlighted the limitations in using EMR for phenotyping such as complex, inaccurate, and missing data problems [27, 28], our approach uses a wide range of clinical features from EMR data and has been shown to work efficiently in both derivation and validation datasets. Our study phenotypes show results consistent with the corresponding MODs. For example, the high mortality of phenotype B (severe hypoxic respiratory failure) is 51%, which is close to the numbers reported (34–46%) in previous studies [3]. Our phenotyping algorithm from EMR data with high richness has been shown to be generalizable and consistent across multiple patient groups from different hospitals, also suggested by prior studies [29, 30].

The unique clinical characteristics of the derived phenotypes and the results of our exploratory analyses are highly informative and can be hypothesis-generating for future research. For example, phenotype A and D appear to suffer from multi-system organ failure (with differences in the organs involved), and may require tailored interventions according to their specific patterns of organ injury. Our results also showed that patients in phenotype A and D were likely to exhibit hyperinflammatory characteristics. However, ARF patients in A had better survival than patients in D overall (Fig. 4). Notably, those who received PEEP ≥ 10 had much better survival than those who did not in phenotype D, but this difference in survival was not better in phenotype A, further suggesting potential differences between the two MOD phenotypes. Phenotype B (severe hypoxemic respiratory failure) is characterized by the most severe degree of hypoxia and associated with the highest mortality. Higher PEEP strategy was also associated with better survival in phenotype B. This could represent the phenotype in which adjunctive treatments for ARDS and severe hypoxemia are needed most often and may be useful for predictive and prognostic enrichment in future clinical trials for ARDS and severe hypoxic respiratory failure. Lastly, phenotype C (mild hypoxia) represents the phenotype with only modest degree of hypoxemia, no other organ injury, and the best outcomes. It was also observed that patients in phenotype C were likely to be hypoinflammatory. We propose that these differences between the phenotypes are worthy of further investigation in future validation studies and clinical trials, and have the potential to provide valuable information regarding potential complications and prognosis, in addition to aid in developing tailored management strategies. Furthermore, the present study phenotypes were identified using early clinical data from the pre-intubation phase of sepsis-induced ARF, which may facilitate prompt classification of patients and candidate selection in future research, as well as timely implementation of tailored management strategies when applied to real clinical settings.

This study has several limitations. First, only routinely collected clinical features in the EMR were used to derive the phenotypes, and integration with other data such as protein biomarkers, clinicians’ impression, immune cell expression or pathogen features during derivation could change assignments of ARF phenotypes. Second, our method relies on the analysis of 24-h pre-intubation data for each patient using median aggregation on each of the features, causing potential loss of temporal information. Third, as the missingness in data was common for some features included in the phenotyping model, MICE-based multivariate imputation strategy was used in the initial analysis. However, features with high missing values were excluded. Fourth, patients who were started on invasive mechanical ventilation (IMV) in the field or in the ED were excluded. Fifth, clinical phenotypes were identified and characterized from a single high-volume integrated health system in the USA with MICU patients. However, a large range of data collection years was considered. Although obtained phenotypes were observed to be generalizable in other hospital system data examined across multiple ICUs, further exploration and extensive validation are required, especially using data from randomized clinical trials, prospective studies, low- and middle-income countries, and longitudinal cohorts.

Our proposed model with explainable artificial intelligence (AI) has an ability to identify important features out of all clinical variables and lab values, which need to be focused specifically to group sepsis-induced ARF patients. The strengths of this study include the usage of large comprehensive datasets from multiple hospitals across multiple ICU, derivation and validation of phenotypes with different hospitals’ data, inclusion of a broad set of routinely collected clinical variables, and mapping of MOD, ARF severity, and mortality outcomes. The consistency in characteristics of the phenotypes in the validation datasets supports the generalizability and reproducibility of our results. Thus, the proposed derivation pipeline for patients with sepsis-induced ARF was found helpful in identifying unique and potentially unclear patterns as well as patient characteristics that can then be utilized both for clinical management and for future research.

In conclusion, we have derived novel phenotypes of sepsis-induced ARF with distinct clinical characteristics and outcomes. The phenotypes are associated with distinct patterns of organ injury, such as cardiac/renal dysfunction, hepatic dysfunction and coagulopathy, and severe hypoxemic respiratory failure resembling ARDS. The phenotypes also demonstrated potential differences in treatment responses to common clinical interventions for sepsis and ARF. Our method can offer valuable knowledge into the diversity of ARF patients with regard to their clinical presentation, prognosis and likelihood of additional complications, which may have a significant impact on the development of tailored management strategies, discussions about goals of care, and patient selection for future clinical trials.

## Supplementary Information

Supplementary Material 1.

## Data Availability

Data and materials might be available upon request. No datasets were generated or analysed during the current study.
