# Supplementary material for: Derivation and validation of generalized sepsis-induced acute respiratory failure phenotypes among critically ill patients: a retrospective study
Source: Crit Care. 2024 Oct 1;28:321. doi: 10.1186/s13054-024-05061-4 (PMC11445942; doi:10.1186/s13054-024-05061-4)
Supplement: Supplementary file 1 — Supplementary Material 1. [file 13054_2024_5061_MOESM1_ESM.docx]

**Derivation and Validation of Generalized Sepsis-induced Acute Respiratory Failure Phenotypes Among Critically Ill Patients: A Retrospective Study**

Tilendra Choudhary (PhD), Pulakesh Upadhyaya (PhD), Carolyn M. Davis (MD), Philip Yang (MD, MSc), Simon Tallowin (BMBS, MSc), Felipe A. Lisboa (MD), Seth A. Schobel (PhD), Craig M. Coopersmith (MD), Eric A. Elster (MD), Timothy G. Buchman (MD), Christopher J. Dente (MD), Rishikesan Kamaleswaran (PhD)

**Online Data Supplement Information**

**Appendix-1.** Characterization of hyper-/hypo-inflammatory nature of the derived phenotypes .................... Page 3

**A1.1.** Method of Validating the Sepsis-induced ARF Phenotypes Against Hyper/Hypo Inflammatory ARDS Subtypes ……………………………………………………………………………...……………… Page 3

**A1.2.** Results of Validating the phenotypes against the Hyper/Hypo Inflammatory Phenotypes

**A1.3.** References ……………………………………………………………………………….…..... Page 3

**Appendix-2:** Methodology and Interpretation of SHAP Plotting .................................................................... Page 4

**Supplemental Table E1.** Summary of electronic medical record (EMR)-based clinical variables or features (f1-f50) used in our study. …………………………………………………………………………………………….. Page 4

**Supplemental Table E2.** Clustering performance with different dimension reduction schemes on yielding various number of clusters. …………………………………………………………………………………………… Page 5

**Supplemental Figure E1.** A bar plot showing mean square error (MSE) between the reconstructed data from the UMAP embedding and the original data for different UMAP dimensions. …………………………………. Page 5

**Supplemental Table E3.** Highlighting the most-frequently occurred diseases per phenotype, obtained from diagnosis codes. ……………………………………………………………………………………………… Page 6

**Supplemental Figure E2.** Radar diagrams showing distribution variations of normalized clinical features (f1-f50) by their average values across different phenotypes of the derivation and validation data. Meanings of feature labels (f1-f50) are exactly same as listed in Table E1. ………………………………………………………………….. Page 7

**Supplemental Figure E3.** Radar diagrams showing variability in demographic variables (age, sex, race, ethnicity) and mortality outcomes (in %) across different phenotypes of the derivation and validation data. …………..Page 8

**Supplemental Figure E4.** Shapley Additive exPlanations (SHAP) beeswarm plots highlighting the dominant features to characterize each of the four clusters as phenotypes. It presents SHAP plots for (a) phenotype A, (b) phenotype B, (c) phenotype C, and (d) phenotype D. These plots provide a visual representation of the influence exerted by various features on identifying specific phenotype, illustrating both the magnitude and direction of that influence. SHAP>0 region shows important features that are characterizing a specific phenotype (e.g., right portion of SHAP=0 line in (a) represents membership of features and their distributions for phenotype A), whereas <0 highlights memberships for all other phenotypes. Feature values are color coded here, and the intensity is shown via color bar. …….… Page 9

**Supplemental Figure E5.** Performance of ML classifiers built on the derivation data for predicting multiclass phenotypes showing ROC curves (upper panel) and precision-recall (PR) curves (lower panel). ML classifiers used were logistic regression (LR), random forest, support vector machine (SVM with RBF kernel), and Gaussian naive bayes (from left to right column). Area under the ROC curves (AUROC or AUC) and area under the PR curves (AUPRC) were used to evaluate and compare the model performance. With the highest AUROC and AUPRC, LR model outperformed others. Highest AUROC values (≥0.98 for all classes) achieved by the LR model indicates the best overall performance among others at discriminating the positive and negative classes for all thresholds. Whereas the highest AUPRC values (≥0.92 for all classes) attained by the LR demonstrates the best performance among others on the positive classes, which is most important in imbalanced datasets. …………………………………. Page 10

**Supplemental Figure E6.** Performance of best performing model logistic regression for phenotype prediction in terms of (a) normalized confusion matrix, and (b) classification metrics. The confusion matrix and the classification metrics were evaluated for the randomly chosen 20% test-portion of the derivation set. Class labels shown in (a) have the following mappings: 0: A, 1: B, 2: C, 3: D. Four evaluation metrics namely, precision, recall, F1-score and accuracy were used. …………………………………………………………………………………………………… Page 11

**Supplemental Table E4.** Modeling parameters for building logistic regression model for phenotype prediction. ………………………………………………………………………………………………………………... Page 11

**Supplemental Table E5.** Trained logistic regression model parameters (weights and biases) for phenotype prediction. ……………………………………………………………………………………………………. Page 12

**Supplemental Table E6.** Summary of patient characteristics of the validation cohort (Emory SICU) and its phenotypes. ………………………………………………………………………………………………...… Page 13

**Supplemental Table E7.** Summary of patient characteristics of the validation cohort (Grady SICU) and its phenotypes. …………...……………………………………………………………………………………… Page 14

**Supplemental Figure E7.** UMAP representation of the derivation data showing distribution of patient encounters from 2020-21 (COVID-19 years; orange colored) is same as patients from other study years (blue colored). . Page 15

**Supplemental Table E8.** Distribution of COVID-19 patients in phenotypes across all ICU datasets. ……… Page 15

**Supplemental Table E9.** Maximum individual SOFA per phenotype in 24-hour pre-intubation region for Emory MICU data. …………………………………………………………………………………………………… Page 16

**Supplemental Table E10.** Maximum individual SOFA per phenotype in 24-hour pre-intubation region for Grady MICU data. …………………………………………………………………………………………………… Page 17

**Supplemental Table E11.** Maximum individual SOFA per phenotype in 24-hour pre-intubation region for Emory SICU data. ………………………………………………….……………………………………………..…. Page 18

**Supplemental Table E12.** Maximum individual SOFA per phenotype in 24-hour pre-intubation region for Grady SICU data. ………………………………………..…………………….………………………………..….. Page 19

**Supplemental Table E13.** List of confounders used for propensity score matching (PSM) ……………….. Page 20

**Supplemental Table E14.** Characteristics of treatment analysis for high PEEP regime. ……………...…… Page 20

**Supplemental Figure E8.** Effect size for phenotype A of the derivation set. ………………………………. Page 21

**Supplemental Figure E9.** Effect size for phenotype B of the derivation set. ………………………………. Page 22

**Supplemental Figure E10.** Effect size for phenotype C of the derivation set. ………………………….….. Page 23

**Supplemental Figure E11.** Effect size for phenotype D of the derivation set. …………………………..…. Page 24

**Appendix-1. Characterization of hyper-/hypo-inflammatory nature of the derived phenotypes**

**A1.1. Method of Validating the Sepsis-induced ARF Phenotypes Against Hyper/Hypo Inflammatory ARDS Subtypes:** To investigate how the contributed phenotypes compare to the existing work in the field, we sought to compare the proposed sepsis-induced ARF phenotypes to the ARDS hyper- and hypo-inflammatory subtypes. Calfee and colleagues investigated clinical and biological data to elucidate two phenotypes in various ARDS cohorts, namely hyper- and hypoinflammatory [1–3]. In a recent extension of these approaches, Sinha et al. proposed a clinical only-model that provided robust discrimination of the ARDS cohort into the two relevant subtypes [1]. However, due to the unavailability of the pre-trained models and labeled data contributed by those groups, we were unable to directly evaluate the classifier against our own data, thus we developed a pragmatic alternative, which utilized the value-intervals of their class-defining features for further heuristic characterization. We report the specific statistical dichotomization of the hyper and hypo-inflammatory subtypes based on the ranges of each variable as provided by Sinha et al. in the supplemental documents S2-S6 [1,4].

**A1.2. Results of Validating the phenotypes against the Hyper/Hypo Inflammatory Phenotypes:** We further sought to investigate how the phenotypes derived from the results above compared to the binarized phenotypes, namely the hyperinflammatory and hypoinflammatory phenotypes [1,2]. By comparing the clinical values reported, our results suggested that patients in phenotype A (MOD-1) and D (MOD-2) were most likely associated with hyperinflammation characterized by high values of total bilirubin (mean A:1.4, D:4.8 mg/dL) and creatinine (mean A:4.3, D:2 mg/dL), and low values of platelet count (mean A:191, D:147 ×10^3^/µL), bicarbonate (mean A:22.4, D:20.7 mmol/L), PaCO2 (mean A:38.6, D:34.2 mmHg) and hemoglobin (mean A:9.2, D:9 g/dL), which were consistent with the values of these markers in the hyperinflammatory subphenotype from the previous works [1,2]. Phenotype B with severe hypoxemic respiratory failure demonstrated features that were consistent with neither hyper- nor hypo-inflammatory phenotype, suggesting that this phenotype could either consist of a mix of both phenotypes or represent a completely novel phenotype. On the contrary, patients in C were associated with hypoinflammatory characteristics with lowest values of total bilirubin (mean:1.1 mg/dL) and creatinine (mean:1.4 mg/dL), and highest values of platelet count (mean:231×103/µL), bicarbonate (mean:26 mmol/L), PaCO2 (mean:42.5 mmHg) and hemoglobin (mean:11.6 g/dL). In comparison to the earlier works, the patient population and variables included were not the same. For example, we did not use biologically derived features such as interleukin-6/8 and intercellular adhesion molecule 1. Hence, this characterization of hyper/hypo-inflammatory subgroups in our identified sepsis-induced ARF phenotypes needs further investigation.

**A1.3. References**

[1] Sinha P, Delucchi KL, Chen Y, Zhuo H, Abbott J, Wang C, et al. Latent class analysis-derived subphenotypes are generalisable to observational cohorts of acute respiratory distress syndrome: a prospective study. Thorax. 2022;77:13–21.

[2] Maddali MV, Churpek M, Pham T, Rezoagli E, Zhuo H, Zhao W, et al. Validation and utility of ARDS subphenotypes identified by machine-learning models using clinical data: an observational, multicohort, retrospective analysis. Lancet Respir Med. 2022;10:367–77.

[3] Sinha P, Calfee CS. Phenotypes in acute respiratory distress syndrome. Curr Opin Crit Care. 2019;25:12–20.

[4] Sinha P, Calfee CS, Delucchi KL. Practitioner’s guide to latent class analysis: Methodological considerations and common pitfalls. Crit Care Med. 2021;49:e63–79.

**Appendix-2. Methodology and Interpretation of SHAP Plotting**

SHapley Additive exPlanation (SHAP) is a game theory-based scheme to explain the predicted output of any kind of machine learning model. In our study, for each specific class, we first created a binary membership label array for that class. For example, for the creation of phenotype A class label, if any sample comes under ‘A’, the label will be 1, and for all other class membership of that sample, the label will be 0. In this way, four different binary labels were created corresponding to the four classes. Then, we properly trained a random forest regressor model to predict the binary labels (one specific class vs other classes) for each of the classes. Scaled feature data with 50 clinical variables was used as an input to the model. It is worth noting that the model outputs were numeric fractional and none of the included features was categorical. The model and data were then used to calculate SHAP values, which explain the fractional model outputs. With SHAP values, beeswarm plots were plotted in Figure E4 which highlight the dominant features to characterize each of the four clusters as phenotypes. The plots provide a visual representation of the influence exerted by various features on identifying specific phenotype, illustrating both the magnitude and direction of that influence. SHAP>0 region shows important features that are characterizing a specific phenotype (e.g., right portion of SHAP=0 line in (a) represents membership of features and their distributions for phenotype A), whereas <0 highlights memberships for all other phenotypes. Feature values are color coded also, and the intensity is shown via color bar.

**Supplemental Table E1.** Summary of electronic medical record (EMR)-based clinical variables or features (f1-f50) used in our study.

| **List of routine clinical variables selected in the study: variable names and their meanings** | |
| --- | --- |
| f1 - temperature: Body temperature (^o^C)  f2 - sbp_cuff: Cuff-based systolic blood pressure (mmHg)  f3 - dbp_cuff: Cuff-based diastolic blood pressure (mmHg)  f4 - pulse: Pulse rate (beats per minute)  f5 - unassisted_resp_rate: Respiratory rate (breaths/min.)  f6 - spo2: Blood saturated oxygen concentration, SpO_2_ level (%)  f7 - end_tidal_co2: End-tidal CO_2_ (mmHg)  f8 - o2_flow_rate: (L/minute)  f9 - bicarb_(hco3): (mmol/L)  f10 - blood_urea_nitrogen_(bun): (mg/dL)  f11 - calcium (mg/dL)  f12 - calcium_ionized (mg/dL)  f13 - chloride (mEq/L)  f14 - creatinine: (mg/dL)  f15 - glucose (mmol/L)  f16 - magnesium (mg/dL)  f17 - osmolarity (mOsm/kg)  f18 - phosphorus (mg/dL)  f19 - potassium (mEq/L)  f20 - sodium (mEq/L)  f21 - hemoglobin: (g/dL)  f22 - met_hgb: (g/dL)  f23 - platelets: (×10^9^/L)  f24 - white_blood_cell_count: (×10^9^/L)  f25 - carboxy_hgb (%) | f26 - alanine_aminotransferase_(alt) (U/L)  f27 - albumin: (g/L)  f28 - alkaline_phosphatase (IU/L)  f29 - ammonia: (µg/dL)  f30 - bilirubin_direct: (mg/dL)  f31 - bilirubin_total: (mg/dL)  f32 - fibrinogen: (mg/dL)  f33 - inr: International Normalized Ratio  f34 - lactate_dehydrogenase: (IU/L)  f35 - lactic_acid: (mmol/L)  f36 - partial_prothrombin_time_(ptt): (s)  f37 - protein: (g/dL)  f38 - lipase: (U/L)  f39 - b-type_natriuretic_peptide_(bnp): B-type natriuretic peptide (pg/ml)  f40 - troponin: (ng/mL)  f41 - fio2: Fraction of inspired oxygen (range: 0-1)  f42 - partial_pressure_of_carbon_dioxide_(paco2):  PaCO_2_ (mmHg)  f43 - partial_pressure_of_oxygen_(pao2): PaO_2_ (mmHg)  f44 - ph  f45 - saturation_of_oxygen_(sao2) (%)  f46 - d_dimer: (ng/mL)  f47 - hemoglobin_a1c (%)  f48 - best_map: Mean arterial pressure (mmHg)  f49 - pf_sp: SpO2/FiO2 ratio  f50 - pf_pa: PaO2/FiO2 ratio (mmHg) |

**Supplemental Table E2.** Clustering performance with different dimension reduction schemes on yielding various number of clusters.

| **Method** | **#Clusters: 3** | **#Clusters: 4** | **#Clusters: 5** | **#Clusters: 6** |
| --- | --- | --- | --- | --- |
| UMAP (d=2) | SS=0.406  DB=0.872  CH=3056.52 | **SS=0.418**  **DB=0.786**  **CH=3516.74** | SS=0.398  DB=0.843  CH=3345.24 | SS=0.408  DB=0.782  CH=3430.39 |
| UMAP (d=3) | SS=0.363  DB=1.004  CH=2415.87 | SS=0.358  DB=1.047  CH=2367.67 | SS=0.363  DB=0.964  CH=2265.19 | SS=0.348  DB=0.981  CH=2203.52 |
| UMAP (d=4) | SS= 0.342  DB=1.065  CH=2252.53 | SS=0.328  DB=1.152  CH=2102.55 | SS=0.333  DB=1.078  CH=1973.45 | SS=0.319  DB=1.103  CH=1886.44 |
| UMAP (d=5) | SS=0.33  DB=1.116  CH=2133.4 | SS=0.315  DB=1.228  CH=1950.87 | SS=0.325  DB=1.14  CH=1855.18 | SS=0.315  DB=1.172  CH=1758.35 |
| PCA (v=0.95, d=30) | SS=0.093  DB=2.637  CH=333.14 | SS=0.089  DB=2.622  CH=282.79 | SS=0.058  DB=2.694  CH=244.10 | SS=0.066  DB=2.682  CH=218.78 |
| PCA (v=0.90, d=25) | SS=0.098  DB=2.567  CH=351.62 | SS=0.094  DB=2.536  CH=299.22 | SS=0.062  DB=2.614  CH=258.72 | SS=0.063  DB=2.668  CH=231.69 |
| PCA (v=0.85, d=20) | SS=0.104  DB=2.467  CH=380.10 | SS=0.101  DB=2.428  CH=324.66 | SS=0.069  DB=2.506  CH=281.42 | SS=0.076  DB=2.505  CH=253.07 |
| PCA (v=0.80, d=17) | SS=0.11  DB=2.392  CH=405.69 | SS=0.107  DB=2.341  CH=347.68 | SS=0.075  DB=2.415  CH=302.12 | SS=0.084  DB=2.399  CH=272.05 |
| Abbreviations used — SS: silhouette score, DB: Davies-Bouldin score, CH: Calinksi-Harabasz score, d: dimension, v: variance explained, #Clusters: number of clusters, PCA: principal component analysis. | | | | |


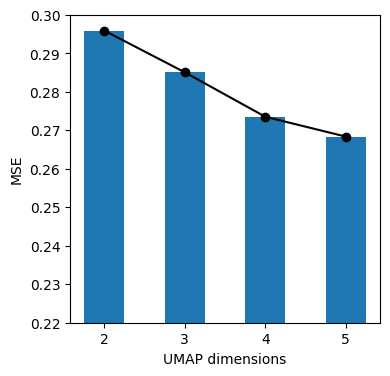


**Supplemental Figure E1.** A bar plot showing mean square error (MSE) between the reconstructed data from the UMAP embedding and the original data for different UMAP dimensions.

**Supplemental Table E3.** Highlighting the most-frequently occurred diseases per phenotype, obtained from diagnosis codes.

| **Phenotype** | **Diagnosis overall** |
| --- | --- |
| A | 'Acute respiratory failure', 'Acute renal failure', 'End stage renal disease', 'Other nonspecific abnormal finding of lung field', 'Other specified forms of effusion, except tuberculous', 'Unspecified septicemia', 'Pulmonary collapse', 'Septic shock', 'Atrial fibrillation', 'Renal dialysis status', 'Unspecified essential hypertension', 'Hyperpotassemia', 'Pneumonia, organism unspecified', 'Coronary atherosclerosis of native coronary artery', 'Other specified cardiac dysrhythmias', 'Cardiac arrest', 'Acute and chronic respiratory failure', 'Pulmonary congestion and hypostasis', 'Hypotension, unspecified', 'Chronic kidney disease, unspecified', 'Unspecified bacteremia', 'Anemia in end-stage renal disease', 'Cardiogenic shock', 'Shortness of breath', 'Congestive heart failure, unspecified', 'Other dyspnea and respiratory abnormality', 'Acute on chronic systolic heart failure', 'Urinary tract infection, site not specified', 'Other shock without mention of trauma', 'Thrombocytopenia, unspecified', 'Cardiomegaly', 'Acute renal failure with lesion of tubular necrosis', 'Pneumonitis due to inhalation of food or vomitus', 'covid-19' |
| B | 'Acute respiratory failure', 'Other nonspecific abnormal finding of lung field', 'Pneumonia, organism unspecified', 'Unspecified septicemia', 'Other specified forms of effusion, except tuberculous', 'Septic shock', 'Unspecified essential hypertension', 'Shock, unspecified', 'Pulmonary collapse', 'Other pulmonary insufficiency, not elsewhere classified', 'Hypoxemia', 'Shortness of breath' 'Pneumonia due to other virus not elsewhere classified', 'Acute and chronic respiratory failure', 'Hyperpotassemia', 'Other dyspnea and respiratory abnormality', 'Pulmonary congestion and hypostasis', 'Cardiac arrest', 'Other fluid overload', 'Other pneumothorax', 'Other shock without mention of trauma', 'Hyperosmolality and/or hypernatremia', 'Other pulmonary embolism and infarction', 'Pneumonitis due to inhalation of food or vomitus', 'Leukocytosis, unspecified', 'Dysphagia, unspecified', 'Fever, unspecified', 'Hypopotassemia', 'covid-19' |
| C | 'Acute respiratory failure', 'Other nonspecific abnormal finding of lung field', 'Other specified forms of effusion, except tuberculous', 'Unspecified septicemia', 'Pulmonary collapse', 'Pneumonia, organism unspecified', 'Unspecified essential hypertension', 'Acute and chronic respiratory failure', 'Septic shock', 'Pneumonitis due to inhalation of food or vomitus', 'Shortness of breath', 'Other dyspnea and respiratory abnormality', 'Shock, unspecified', 'Fever, unspecified', 'Hyperosmolality and/or hypernatremia', 'Diabetes mellitus without mention of complication, type II or unspecified type, not stated as uncontrolled', 'Pulmonary congestion and hypostasis', 'Hypopotassemia', 'Cardiogenic shock', 'Dysphagia, unspecified', 'Unspecified bacteremia', 'Hypoxemia', 'Leukocytosis, unspecified', 'Chronic respiratory failure', 'Other fluid overload', 'Hypotension, unspecified', 'Obstructive chronic bronchitis with (acute) exacerbation', 'covid-19' |
| D | 'Acute respiratory failure', 'Other nonspecific abnormal finding of lung field', 'Unspecified septicemia', 'Other specified forms of effusion, except tuberculous', 'Septic shock', 'Pulmonary collapse', 'Acidosis', 'Shock, unspecified', 'Thrombocytopenia, unspecified', 'Atrial fibrillation', 'Pneumonia, organism unspecified', 'Anemia, unspecified', 'Hepatic coma', 'End stage renal disease', 'Unspecified essential hypertension', 'Acute posthemorrhagic anemia', 'Other specified cardiac dysrhythmias', 'Cirrhosis of liver without mention of alcohol', 'Hypopotassemia', 'Hemorrhage of gastrointestinal tract, unspecified', 'Unspecified bacteremia', 'Alcoholic cirrhosis of liver', 'Acute and subacute necrosis of liver', 'Hypotension, unspecified', 'Fever, unspecified', 'Liver replaced by transplant', 'Shortness of breath', 'Other dyspnea and respiratory abnormality', 'Pulmonary congestion and hypostasis', 'Other shock without mention of trauma', 'Edema', 'Pneumonitis due to inhalation of food or vomitus', ‘Coronary atherosclerosis of native coronary artery', 'Leukocytosis, unspecified', 'Other pancytopenia', 'covid-19' |


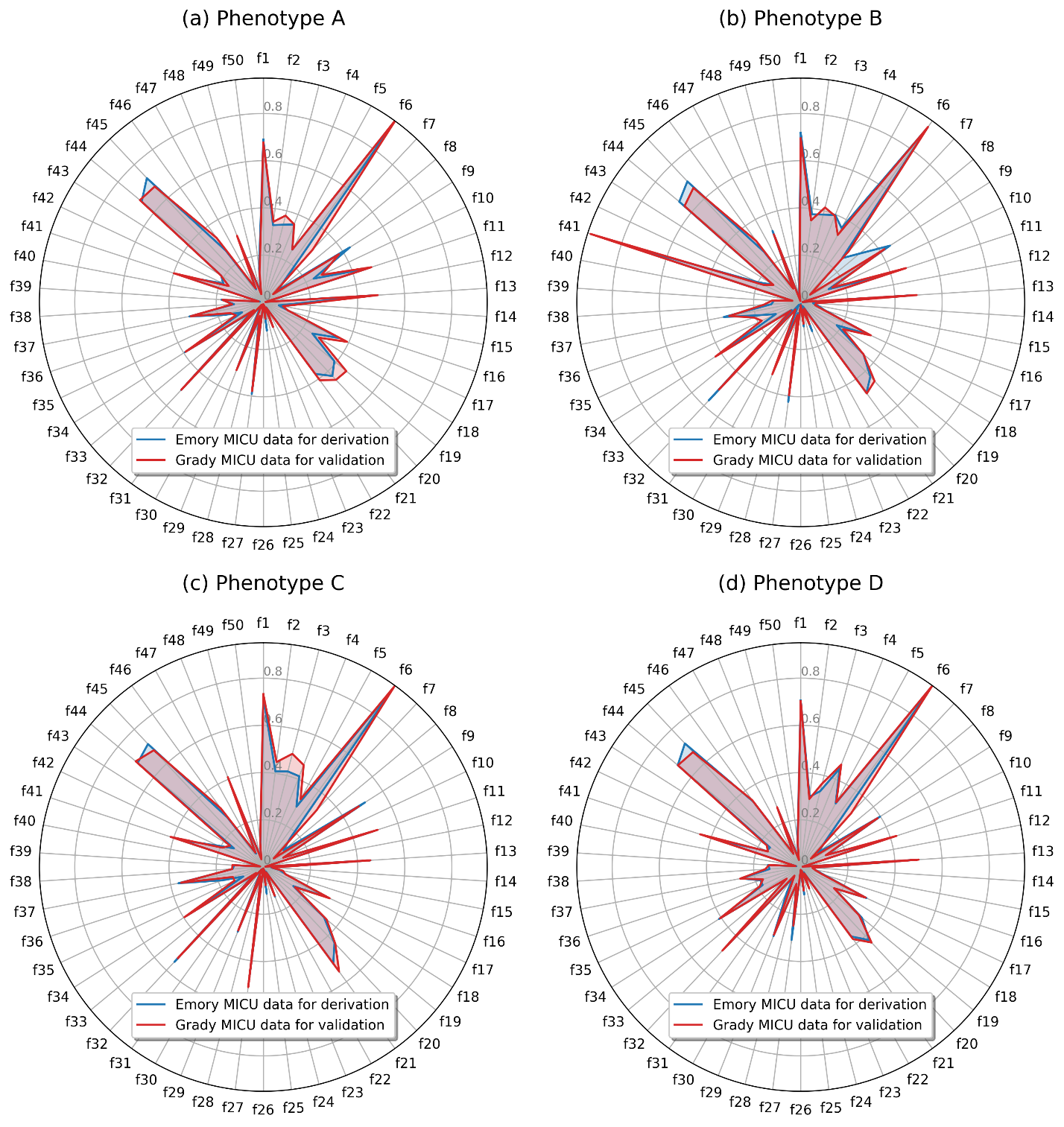


**Supplemental Figure E2.** Radar diagrams showing distribution variations of normalized clinical features (f1-f50) by their average values across different phenotypes of the derivation and validation data. Meanings of feature labels (f1-f50) are exactly same as listed in Table E1.


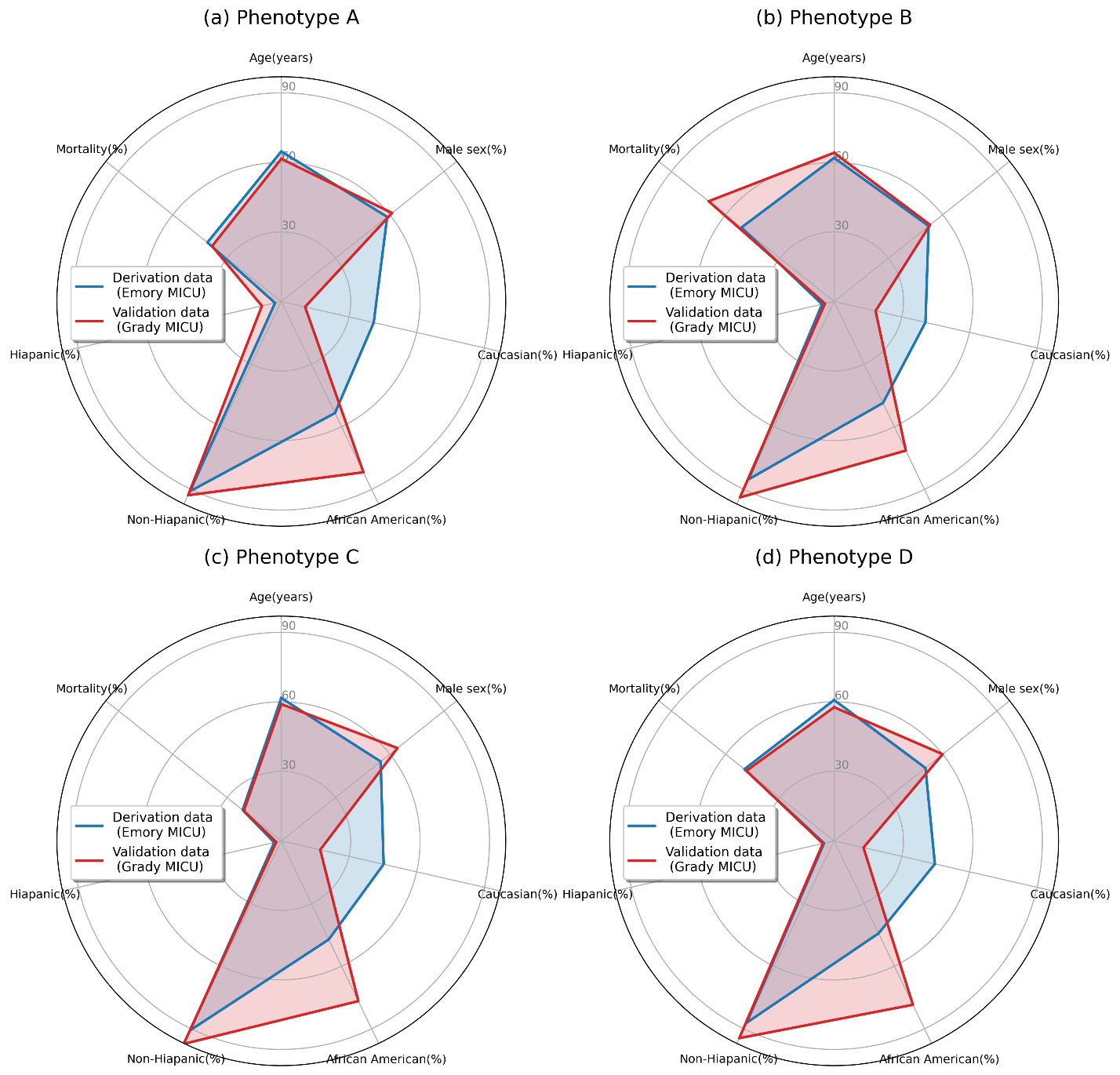


**Supplemental Figure E3.** Radar diagrams showing variability in demographic variables (age, sex, race, ethnicity) and mortality outcomes (in %) across different phenotypes of the derivation and validation data


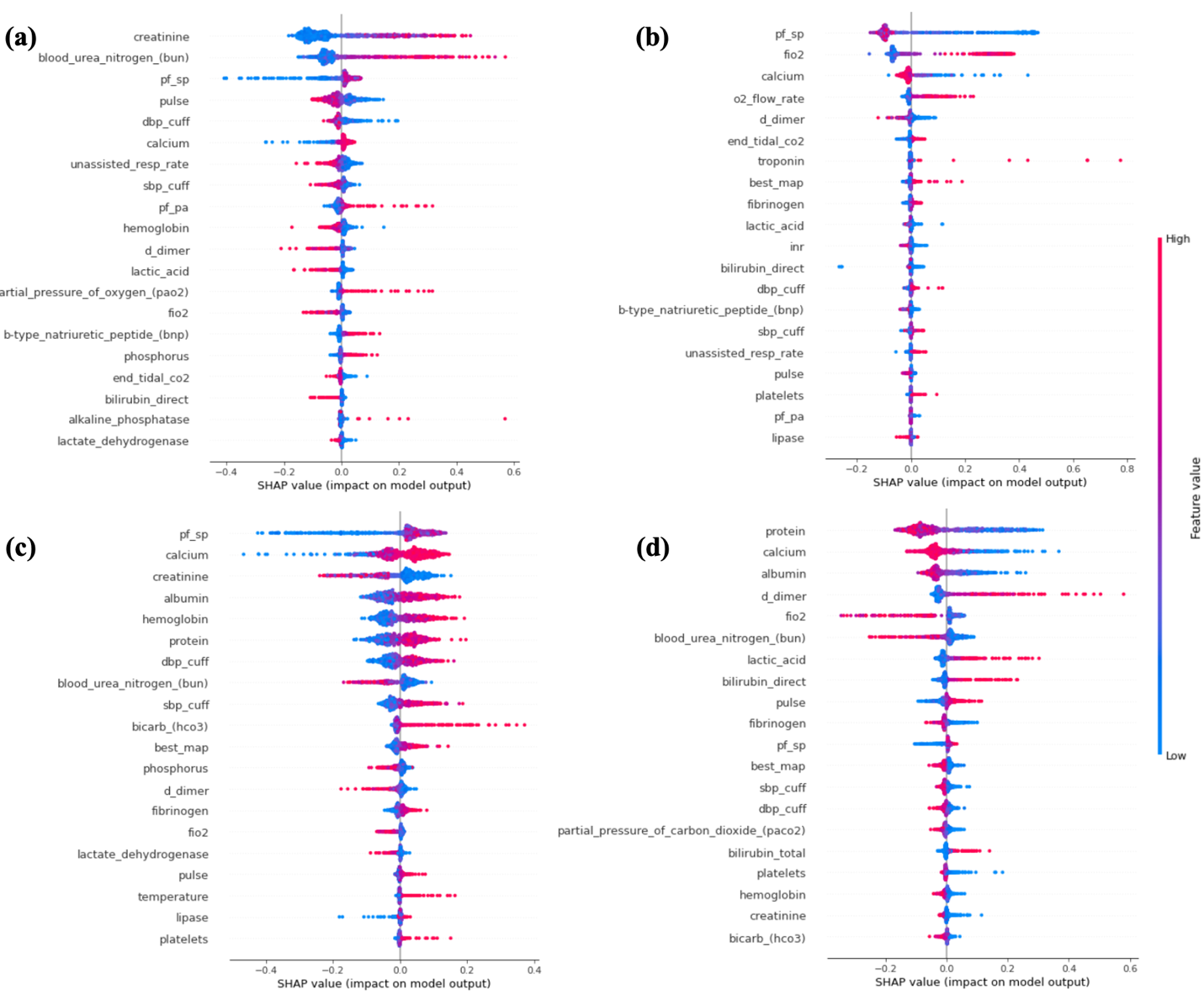


**Supplemental Figure E4.** Shapley Additive exPlanations (SHAP) beeswarm plots highlighting the dominant features to characterize each of the four clusters as phenotypes. It presents SHAP plots for (a) phenotype A, (b) phenotype B, (c) phenotype C, and (d) phenotype D. These plots provide a visual representation of the influence exerted by various features on identifying specific phenotype, illustrating both the magnitude and direction of that influence. SHAP>0 region shows important features that are characterizing a specific phenotype (e.g., right portion of SHAP=0 line in (a) represents membership of features and their distributions for phenotype A), whereas <0 highlights memberships for all other phenotypes. Feature values are color coded here, and the intensity is shown via color bar.


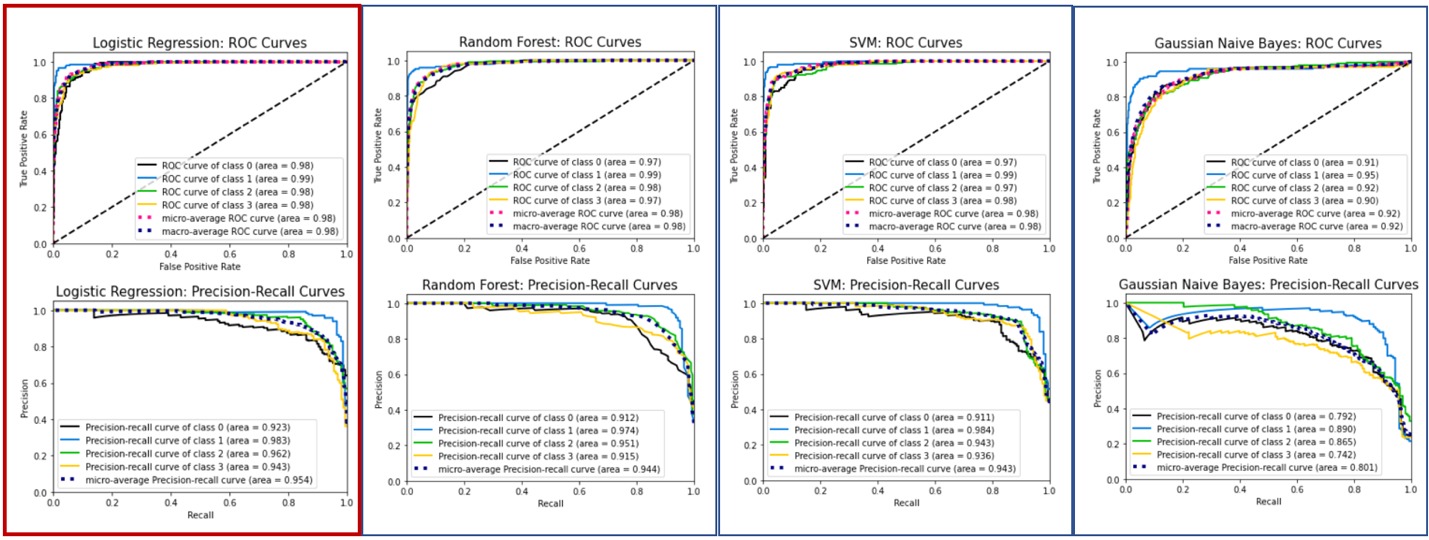


**Supplemental Figure E5.** Performance of ML classifiers built on the derivation data for predicting multiclass phenotypes showing ROC curves (upper panel) and precision-recall (PR) curves (lower panel). ML classifiers used were logistic regression (LR), random forest, support vector machine (SVM with RBF kernel), and Gaussian naive bayes (from left to right column). Area under the ROC curves (AUROC or AUC) and area under the PR curves (AUPRC) were used to evaluate and compare the model performance. With the highest AUROC and AUPRC, LR model outperformed others. Highest AUROC values (≥0.98 for all classes) achieved by the LR model indicates the best overall performance among others at discriminating the positive and negative classes for all thresholds. Whereas the highest AUPRC values (≥0.92 for all classes) attained by the LR demonstrates the best performance among others on the positive classes, which is most important in imbalanced datasets.


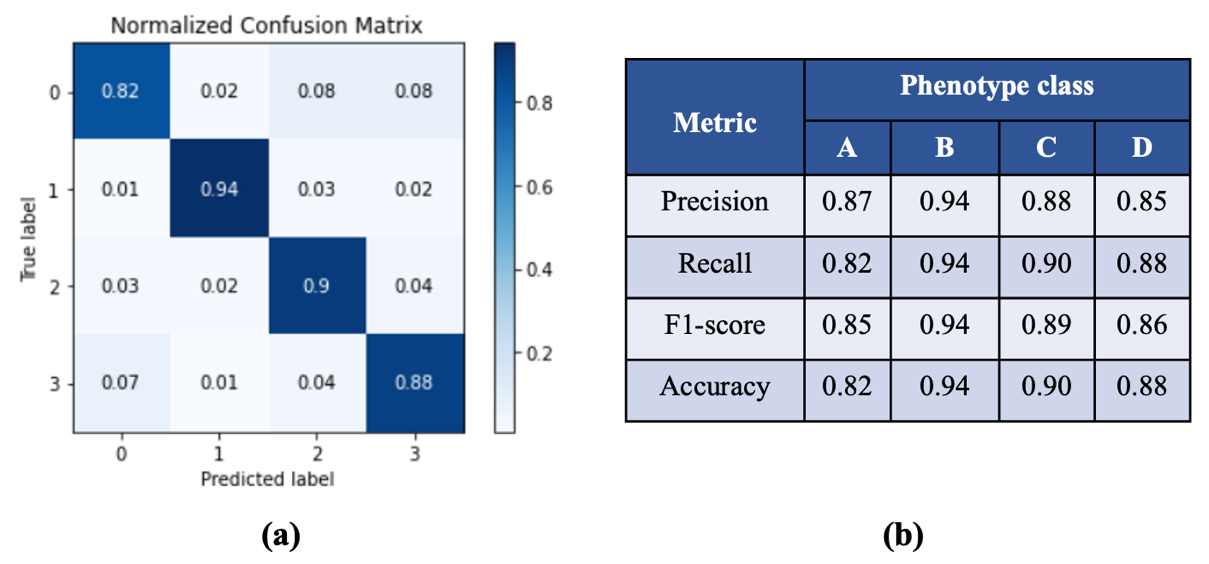


**Supplemental Figure E6.** Performance of best performing model logistic regression for phenotype prediction in terms of (a) normalized confusion matrix, and (b) classification metrics. The confusion matrix and the classification metrics were evaluated for the randomly chosen 20% test-portion of the derivation set. Class labels shown in (a) have the following mappings: 0: A, 1: B, 2: C, 3: D. Four evaluation metrics namely, precision, recall, F1-score and accuracy were used.

**Supplemental Table E4.** Modeling parameters for building logistic regression model for phenotype prediction.

| **Parameters** | **Description** |
| --- | --- |
| Model name | Multinomial logistic regression |
| Input data | Scaled multivariate feature data with 50 features (derivation set) |
| Number of features | 50 |
| Output classes | Four phenotypes (A, B, C, D) |
| Penalty | L2 |
| Solver | lbfgs |
| Maximum iterations | 100 |

**Supplemental Table E5.** Trained logistic regression model parameters (weights and biases) for phenotype prediction.

| **Parameter** | **Feature** | **Class (Phenotype)** | | | |
| --- | --- | --- | --- | --- | --- |
|  |  | **A** | **B** | **C** | **D** |
| **Weights** | temperature | -0.8942 | 0.1902 | 0.4677 | 0.2362 |
|  | sbp_cuff | -1.5247 | 1.1100 | 1.9926 | -1.5779 |
|  | dbp_cuff | -1.3127 | 0.7708 | 2.1643 | -1.6224 |
|  | pulse | -2.2834 | -0.8696 | 0.7544 | 2.3986 |
|  | unassisted_resp_rate | -2.3979 | 2.1731 | -0.1628 | 0.3876 |
|  | spo2 | -0.2301 | -0.4867 | 0.1349 | 0.5820 |
|  | end_tidal_co2 | -1.6346 | 2.2576 | -0.9103 | 0.2873 |
|  | o2_flow_rate | -0.2156 | 1.0959 | -0.7759 | -0.1044 |
|  | bicarb_(hco3) | -0.2951 | -0.5812 | 2.3449 | -1.4686 |
|  | blood_urea_nitrogen_(bun) | 4.5446 | -0.5196 | -2.1935 | -1.8314 |
|  | calcium | 2.9377 | -3.4623 | 4.3886 | -3.8640 |
|  | calcium_ionized | 0.0022 | -0.1167 | 0.1228 | -0.0083 |
|  | chloride | -0.5607 | 0.2645 | -0.4806 | 0.7768 |
|  | creatinine | 4.5741 | -0.3095 | -3.1605 | -1.1041 |
|  | glucose | -0.4907 | -0.2748 | 0.9329 | -0.1674 |
|  | magnesium | 0.7473 | 0.3282 | -0.3637 | -0.7118 |
|  | osmolarity | 1.7403 | -0.2515 | -0.2827 | -1.2060 |
|  | phosphorus | 2.7643 | -0.1448 | -2.0321 | -0.5873 |
|  | potassium | 1.3852 | -0.2484 | -0.2772 | -0.8597 |
|  | sodium | 0.0000 | 0.2103 | 0.2848 | -0.4951 |
|  | hemoglobin | -2.0269 | 1.4456 | 2.9995 | -2.4182 |
|  | met_hgb | -0.2542 | -0.1770 | 0.3351 | 0.0961 |
|  | platelets | -0.2929 | 0.8235 | 0.8644 | -1.3950 |
|  | white_blood_cell_count | 0.5639 | 0.0598 | -0.3265 | -0.2971 |
|  | carboxy_hgb | 0.3875 | -0.3721 | 0.0175 | -0.0329 |
|  | alanine_aminotransferase_(alt) | -0.3034 | -0.5397 | 0.3358 | 0.5074 |
|  | albumin | -0.4809 | 0.9134 | 3.1312 | -3.5637 |
|  | alkaline_phosphatase | 1.5187 | -0.7093 | -1.0722 | 0.2628 |
|  | ammonia | -0.4755 | 1.0854 | -0.3197 | -0.2902 |
|  | bilirubin_direct | -1.5618 | 0.1549 | -0.2470 | 1.6539 |
|  | bilirubin_total | -1.6135 | -0.0480 | -0.2027 | 1.8642 |
|  | fibrinogen | -0.7200 | 1.1118 | 1.1540 | -1.5458 |
|  | inr | 0.5967 | -0.9787 | -0.4621 | 0.8441 |
|  | lactate_dehydrogenase | -0.8933 | 0.1871 | -0.5750 | 1.2812 |
|  | lactic_acid | -2.6171 | 0.6205 | -0.5890 | 2.5856 |
|  | partial_prothrombin_time_(ptt) | -0.4854 | -0.1658 | 1.1319 | -0.4807 |
|  | protein | -0.4106 | 0.4159 | 3.3833 | -3.3886 |
|  | lipase | -0.0718 | -0.9683 | 0.8748 | 0.1653 |
|  | b-type_natriuretic_peptide_(bnp) | 2.1336 | -1.3594 | -0.1078 | -0.6664 |
|  | troponin | -1.5409 | 3.0891 | -1.3849 | -0.1633 |
|  | fio2 | -3.5764 | 9.1355 | -2.8194 | -2.7397 |
|  | partial_pressure_of_carbon_dioxide_(paco2) | -0.0155 | -0.1184 | 0.4539 | -0.3200 |
|  | partial_pressure_of_oxygen_(pao2) | 3.5136 | -0.5709 | -1.1517 | -1.7909 |
|  | ph | 0.3525 | -0.4227 | 0.0809 | -0.0107 |
|  | saturation_of_oxygen_(sao2) | -0.0139 | -0.2868 | 0.4678 | -0.1671 |
|  | d_dimer | -1.0465 | -2.2670 | -1.4686 | 4.7821 |
|  | hemoglobin_a1c | -0.1736 | -0.1224 | -0.0654 | 0.3613 |
|  | best_map | -1.1940 | 1.2145 | 1.2340 | -1.2546 |
|  | pf_sp | 0.0597 | -0.2167 | 0.1709 | -0.0140 |
|  | pf_pa | 1.9320 | -0.6817 | -0.7717 | -0.4786 |
| **Bias** | – | 4.2119 | -5.7972 | -5.7017 | 7.2870 |

**Supplemental Table E6.** Summary of patient characteristics of the validation cohort (Emory SICU) and its phenotypes.

| **Parameters** | **Whole cohort** | **A** | **B** | **C** | **D** | ***p*-value** |
| --- | --- | --- | --- | --- | --- | --- |
| **count (%)** | 1128 (100) | 818 (72.5) | 39 (3.5) | 259 (23) | 12 (1.1) | - |
| **Mortality*** | 255, 22.93% | 196, 24.32% | 13, 33.33% | 42, 16.22% | 4, 33.33% | - |
| **Age, mean(std)** | 60.4 (14.6) | 61.1 (14.1) | 59.5 (16.9) | 58.8 (15.4) | 49.3 (18.2) | 0.008 |
| **Males, count (%)** | 661 (58.6) | 477 (58.3) | 18 (46.2) | 161 (62.2) | 5 (41.7) | - |
| **Race: African American or Black, count (%)** | 465 (41.2) | 333 (40.7) | 17 (43.6) | 111 (42.9) | 4 (33.3) | 0.948 |
| **Race: Caucasian or White, count (%)** | 560 (49.6) | 407 (49.8) | 20 (51.3) | 126 (48.6) | 7 (58.3) |  |
| **Ethnicity: Hispanic, count (%)** | 34 (3.0) | 22 (2.7) | 2 (5.1) | 10 (3.9) | 0 (0) | 0.848 |
| **Ethnicity: Non-Hispanic, count (%)** | 1011 (89.6) | 739 (90.3) | 34 (87.2) | 227 (87.6) | 11 (91.7) |  |
| **P/F ratio, m(IQR)** | 262.5 [183.5,344.0] | 280.0 [202.5,362.5] | 109.3 [74.1,143.0] | 225.0 [172.4,299.2] | 260.7 [190.0,305.6] | <0.001 |
| **S/F ratio, m(IQR)** | 247.5 [211.1,316.7] | 250.0 [232.5,322.6] | 98.5 [94.9,121.6] | 245.0 [198.9,283.5] | 243.8 [211.3,264.3] | <0.001 |
| **FiO_2_, m(IQR)** | 0.4 [0.3,0.5] | 0.4 [0.3,0.4] | 1.0 [0.8,1.0] | 0.4 [0.3,0.5] | 0.4 [0.4,0.5] | <0.001 |
| **PaO_2_, m(IQR)** | 102.0 [84.0,130.0] | 106.0 [87.0,135.0] | 74.0 [67.5,91.5] | 93.0 [80.0,116.2] | 106.0 [90.0,122.5] | <0.001 |
| **PaCO_2_, m(IQR)** | 38.0 [34.0,43.0] | 38.0 [34.0,42.0] | 36.0 [32.0,44.0] | 39.9 [36.0,46.0] | 33.0 [24.0,39.5] | <0.001 |
| **MAP, m(IQR)** | 83.5 [78.0,91.0] | 82.0 [77.0,89.0] | 88.5 [77.6,95.3] | 88.2 [83.0,97.0] | 76.8 [75.4,80.1] | <0.001 |
| **Creatinine, m(IQR)** | 1.2 [0.8,2.2] | 1.4 [0.9,2.7] | 1.2 [0.8,1.7] | 0.9 [0.7,1.3] | 1.5 [1.0,2.3] | <0.001 |
| **Bilirubin total, m(IQR)** | 0.7 [0.5,1.3] | 0.8 [0.5,1.5] | 0.7 [0.5,1.3] | 0.6 [0.5,0.9] | 1.1 [0.7,3.9] | 0.001 |
| **Albumin, m(IQR)** | 2.8 [2.4,3.3] | 2.7 [2.3,3.2] | 2.8 [2.5,3.3] | 3.4 [2.8,3.8] | 2.2 [1.9,2.7] | <0.001 |
| **Lactic acid, m(IQR)** | 1.5 [1.2,2.0] | 1.5 [1.2,2.0] | 1.9 [1.3,2.4] | 1.5 [1.2,1.8] | 1.9 [1.7,4.2] | 0.005 |
| **D-dimer, m(IQR)** | 3652.0 [1496.8,8418.5] | 4143.0 [1687.0,9999.0] | 2160.5 [1563.0,5248.8] | 2396.8 [1101.8,5710.0] | 7475.0 [2723.5,11287.5] | 0.026 |
| **Platelets, m(IQR)** | 210.5 [139.8,288.0] | 202.0 [130.0,285.1] | 197.0 [148.0,240.0] | 234.0 [176.0,303.5] | 96.5 [46.8,186.2] | <0.001 |
| **Hemoglobin, m(IQR)** | 9.4 [8.2,11.1] | 8.8 [8.0,10.2] | 10.4 [8.8,12.0] | 11.8 [10.2,13.5] | 8.9 [8.4,9.5] | <0.001 |
| **BNP, m(IQR)** | 328.0 [110.5,831.0] | 444.0 [147.6,998.2] | 250.5 [80.8,479.5] | 126.0 [42.0,432.0] | 440.5 [195.2,1190.5] | <0.001 |
| **BUN, m(IQR)** | 25.0 [16.0,40.0] | 29.0 [19.0,47.0] | 29.0 [19.5,44.5] | 17.0 [12.0,23.9] | 14.5 [10.5,27.2] | <0.001 |
| **SOFA max total, m(IQR)** | 7.0 [4.0,9.0] | 7.0 [5.0,9.0] | 7.0 [5.0,9.0] | 5.0 [4.0,8.0] | 11.0 [8.0,12.0] | <0.001 |
| **GCS total score, m(IQR)** | 15.0 [13.0,15.0] | 15.0 [13.0,15.0] | 15.0 [11.0,15.0] | 15.0 [13.0,15.0] | 14.5 [11.5,15.0] | 0.783 |
| **PEEP, m(IQR)** | 6.0 [6.0,8.0] | 6.0 [6.0,8.0] | 10.0 [8.0,12.0] | 6.0 [6.0,10.0] | 7.0 [5.8,8.5] | <0.001 |
| For clinical variables, this table lists the medians and interquartile ranges (IQR: Q1-Q3) for each phenotype as well as for the whole cohort. The p-value is also provided for each variable to indicate the statistical significance of the differences among the phenotypes. For evaluating statistical significance, Kruskal-Wallis test was performed for continuous variables and Chi-squared test was used for categorical variables. *Mortality was computed with respect to patients (not encounters). **Abbreviations used —** count: total encounters, mean: average, std: standard deviation, m: median, IQR: interquartile range, PaO_2_: partial pressure of oxygen, SpO_2_: peripheral oxygen saturation level, FiO_2_: fraction of inspired oxygen, P/F: PaO_2_/FiO_2_ ratio, S/F: SpO_2_/FiO_2_ ratio, PaCO_2_: partial pressure of carbon dioxide in arterial blood, MAP: mean arterial blood pressure, Resp.: respiration, BNP: B-type natriuretic peptide, BUN: blood urea nitrogen, SOFA: sequential organ failure assessment, GCS: Glasgow coma scale. **Measurement units —** P/F ratio, PaO2, PaCO2, and MAP: mmHg; S/F ratio and FiO_2_: unitless; creatinine and bilirubin total: mg/dL; albumin: g/L; lactic acid: mmol/L; D-dimer: ng/mL; platelets: ×10^3^/µL; hemoglobin: g/dL; BNP: pg/mL; BUN: mg/dL. | | | | | | |

**Supplemental Table E7.** Summary of patient characteristics of the validation cohort (Grady SICU) and its phenotypes.

| **Parameters** | **Whole cohort** | **A** | **B** | **C** | **D** | ***p*-value** |
| --- | --- | --- | --- | --- | --- | --- |
| **count (%)** | 466 (100) | 133 (28.5) | 5 (1.1) | 264 (56.7) | 64 (13.7) | - |
| **Mortality*** | 91, 19.57% | 32, 24.06% | 4, 80% | 32, 12.12% | 23, 35.94% | - |
| **Age, mean(std)** | 56.0 (16.4) | 60.2 (15.4) | 61.6 (6.2) | 53.9 (16.1) | 55.4 (18.5) | 0.003 |
| **Males, count (%)** | 284 (60.9) | 74 (55.6) | 1 (20.0) | 175 (66.3) | 34 (53.1) | - |
| **Race: African American or Black, count (%)** | 354 (76.0) | 106 (79.7) | 5 (100.0) | 194 (73.5) | 49 (76.6) | 0.497 |
| **Race: Caucasian or White, count (%)** | 77 (16.5) | 16 (12.0) | 0 (0) | 49 (18.6) | 12 (18.8) |  |
| **Ethnicity: Hispanic, count (%)** | 20 (4.3) | 4 (3.0) | 0 (0) | 12 (4.5) | 4 (6.2) | 0.928 |
| **Ethnicity: Non-Hispanic, count (%)** | 443 (95.1) | 128 (96.2) | 5 (100.0) | 250 (94.7) | 60 (93.8) |  |
| **P/F ratio, m(IQR)** | 305.0 [245.0,365.0] | 334.3 [290.5,382.0] | 94.3 [85.5,202.0] | 292.5 [236.3,357.5] | 284.2 [251.4,333.8] | <0.001 |
| **S/F ratio, m(IQR)** | 250.0 [242.5,250.0] | 250.0 [245.9,267.3] | 100.8 [100.0,104.4] | 250.0 [242.5,250.0] | 249.5 [235.1,250.0] | <0.001 |
| **FiO_2_, m(IQR)** | 0.4 [0.4,0.4] | 0.4 [0.4,0.4] | 0.9 [0.9,1.0] | 0.4 [0.4,0.4] | 0.4 [0.4,0.4] | <0.001 |
| **PaO_2_, m(IQR)** | 121.0 [99.0,144.0] | 137.0 [114.0,151.0] | 79.0 [76.0,100.0] | 114.0 [96.5,141.0] | 111.5 [99.8,127.5] | <0.001 |
| **PaCO_2_, m(IQR)** | 37.0 [33.0,41.0] | 36.0 [31.0,40.0] | 43.0 [35.0,50.0] | 38.0 [34.0,41.0] | 36.5 [30.8,40.0] | 0.008 |
| **MAP, m(IQR)** | 89.0 [82.0,95.5] | 84.8 [77.0,91.0] | 78.0 [77.5,80.0] | 93.0 [87.0,98.0] | 81.0 [76.0,86.6] | <0.001 |
| **Creatinine, m(IQR)** | 1.2 [0.8,2.0] | 2.6 [1.5,5.4] | 1.1 [1.1,1.5] | 1.0 [0.8,1.4] | 1.1 [0.9,1.7] | <0.001 |
| **Bilirubin total, m(IQR)** | 0.7 [0.5,1.3] | 0.6 [0.4,1.2] | 0.8 [0.4,1.4] | 0.7 [0.5,1.2] | 1.1 [0.6,2.3] | 0.001 |
| **Albumin, m(IQR)** | 3.0 [2.4,3.4] | 2.7 [2.3,3.1] | 3.0 [2.6,3.2] | 3.3 [2.8,3.7] | 2.1 [1.9,2.5] | <0.001 |
| **Lactic acid, m(IQR)** | 2.2 [1.7,3.4] | 2.2 [1.6,3.2] | 5.4 [4.4,6.1] | 2.1 [1.6,3.2] | 2.6 [2.0,4.0] | <0.001 |
| **D-dimer, m(IQR)** | 6261.0 [2355.0,13674.0] | 6430.0 [2471.1,12292.2] | 3688.0 [3688.0,3688.0] | 5251.5 [1961.8,20407.0] | 6555.5 [3303.0,14402.1] | 0.812 |
| **Platelets, m(IQR)** | 216.5 [147.0,311.0] | 208.0 [146.0,301.0] | 250.0 [200.0,302.0] | 234.0 [160.8,320.0] | 163.0 [104.2,299.2] | 0.012 |
| **Hemoglobin, m(IQR)** | 10.6 [8.8,12.7] | 9.1 [7.8,10.9] | 10.0 [7.5,10.6] | 11.8 [9.8,13.6] | 9.2 [8.4,10.5] | <0.001 |
| **BNP, m(IQR)** | 209.0 [78.5,589.0] | 334.0 [129.5,1457.8] | 812.0 [477.8,1881.0] | 165.5 [54.0,366.0] | 173.0 [89.0,384.5] | <0.001 |
| **BUN, m(IQR)** | 22.0 [14.0,38.0] | 47.0 [29.0,71.0] | 31.0 [17.0,38.0] | 17.0 [12.0,23.0] | 22.2 [16.0,31.0] | <0.001 |
| **SOFA max total, m(IQR)** | 5.0 [3.0,8.0] | 6.0 [4.0,9.0] | 7.0 [5.0,8.0] | 4.0 [3.0,6.0] | 6.0 [4.0,9.0] | <0.001 |
| **GCS total score, m(IQR)** | 15.0 [13.0,15.0] | 15.0 [13.0,15.0] | 15.0 [15.0,15.0] | 15.0 [12.5,15.0] | 15.0 [14.0,15.0] | 0.543 |
| **PEEP, m(IQR)** | 8.0 [5.0,8.0] | 8.0 [5.0,8.0] | 8.0 [8.0,8.0] | 8.0 [5.0,8.0] | 8.0 [5.0,8.0] | 0.34 |
| For clinical variables, this table lists the medians and interquartile ranges (IQR: Q1-Q3) for each phenotype as well as for the whole cohort. The p-value is also provided for each variable to indicate the statistical significance of the differences among the phenotypes. For evaluating statistical significance, Kruskal-Wallis test was performed for continuous variables and Chi-squared test was used for categorical variables. *Mortality was computed with respect to patients (not encounters). **Abbreviations used —** count: total encounters, mean: average, std: standard deviation, m: median, IQR: interquartile range, PaO_2_: partial pressure of oxygen, SpO_2_: peripheral oxygen saturation level, FiO_2_: fraction of inspired oxygen, P/F: PaO_2_/FiO_2_ ratio, S/F: SpO_2_/FiO_2_ ratio, PaCO_2_: partial pressure of carbon dioxide in arterial blood, MAP: mean arterial blood pressure, Resp.: respiration, BNP: B-type natriuretic peptide, BUN: blood urea nitrogen, SOFA: sequential organ failure assessment, GCS: Glasgow coma scale. **Measurement units —** P/F ratio, PaO2, PaCO2, and MAP: mmHg; S/F ratio and FiO_2_: unitless; creatinine and bilirubin total: mg/dL; albumin: g/L; lactic acid: mmol/L; D-dimer: ng/mL; platelets: ×10^3^/µL; hemoglobin: g/dL; BNP: pg/mL; BUN: mg/dL. | | | | | | |


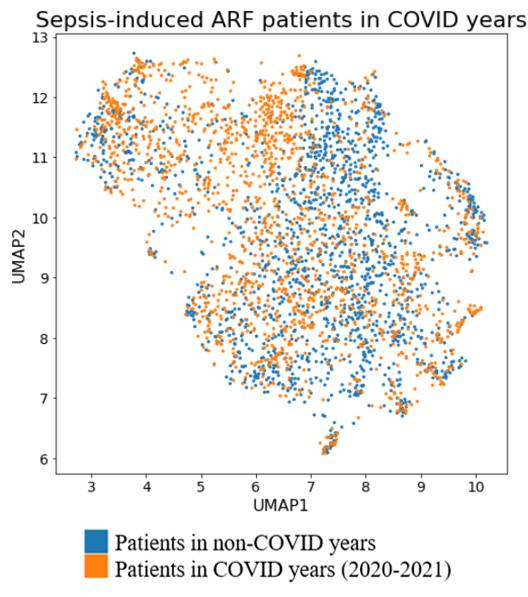


**Supplemental Figure E7.** UMAP representation of the derivation data showing distribution of patient encounters from 2020-21 (COVID-19 years; orange colored) is same as patients from other study years (blue colored).

**Supplemental Table E8.** Distribution of COVID-19 patients in phenotypes across all ICU datasets.

| **Datasets** | **Whole** | **A** | **B** | **C** | **D** |
| --- | --- | --- | --- | --- | --- |
| Emory MICU | 17 (568/3349) | 8.6 (73/845) | 41.8 (289/692) | 13.8 (137/993) | 8.4 (69/819) |
| Grady MICU | 3.5 (30/867) | 2.3 (5/214) | 12.2 (6/49) | 3.7 (15/404) | 2 (4/200) |
| Emory SICU | 6.7 (76/1128) | 5.5 (45/818) | 20.5 (8/39) | 8.9 (23/259) | 0 (0/12) |
| Grady SICU | 1.1 (5/466) | 0.75 (1/133) | 0 (0/5) | 1.5 (4/264) | 0 (0/64) |
| * Distribution of patients with COVID-19 is shown in format: % (n_covid/n_total) | | | | | |

**Supplemental Table E9.** Maximum individual SOFA per phenotype in 24-hour pre-intubation region for Emory MICU data.

| Individual SOFA for Emory MICU | SOFA value | Whole cohort | A | B | C | D | **p-v**alue |
| --- | --- | --- | --- | --- | --- | --- | --- |
| n | - | 3349 | 845 | 692 | 993 | 819 | - |
| SOFA max coagulopathy, n (%) | 0 | 1772 (59.1) | 432 (58.1) | 438 (70.4) | 637 (72.1) | 265 (35.4) | <0.001 |
|  | 1 | 513 (17.1) | 141 (19.0) | 102 (16.4) | 146 (16.5) | 124 (16.6) |  |
|  | 2 | 345 (11.5) | 91 (12.2) | 46 (7.4) | 59 (6.7) | 149 (19.9) |  |
|  | 3 | 225 (7.5) | 59 (7.9) | 17 (2.7) | 24 (2.7) | 125 (16.7) |  |
|  | 4 | 142 (4.7) | 20 (2.7) | 19 (3.1) | 18 (2.0) | 85 (11.4) |  |
| SOFA max renal, n (%) | 0 | 1115 (34.7) | 79 (9.7) | 282 (43.1) | 483 (50.6) | 271 (34.5) | <0.001 |
|  | 1 | 757 (23.6) | 115 (14.1) | 163 (24.9) | 282 (29.6) | 197 (25.1) |  |
|  | 2 | 609 (19.0) | 191 (23.3) | 112 (17.1) | 127 (13.3) | 179 (22.8) |  |
|  | 3 | 296 (9.2) | 139 (17.0) | 39 (6.0) | 39 (4.1) | 79 (10.1) |  |
|  | 4 | 435 (13.5) | 294 (35.9) | 58 (8.9) | 23 (2.4) | 60 (7.6) |  |
| SOFA max hepatic, n (%) | 0 | 1563 (65.0) | 368 (64.7) | 395 (76.7) | 527 (75.3) | 273 (44.0) | <0.001 |
|  | 1 | 332 (13.8) | 86 (15.1) | 68 (13.2) | 95 (13.6) | 83 (13.4) |  |
|  | 2 | 318 (13.2) | 88 (15.5) | 38 (7.4) | 62 (8.9) | 130 (20.9) |  |
|  | 3 | 95 (4.0) | 22 (3.9) | 10 (1.9) | 10 (1.4) | 53 (8.5) |  |
|  | 4 | 97 (4.0) | 5 (0.9) | 4 (0.8) | 6 (0.9) | 82 (13.2) |  |
| SOFA max neuro, n (%) | 0 | 949 (36.4) | 195 (29.8) | 288 (51.4) | 255 (35.3) | 211 (31.6) | <0.001 |
|  | 1 | 564 (21.7) | 157 (24.0) | 107 (19.1) | 146 (20.2) | 154 (23.1) |  |
|  | 2 | 438 (16.8) | 130 (19.9) | 58 (10.4) | 135 (18.7) | 115 (17.2) |  |
|  | 3 | 436 (16.7) | 111 (17.0) | 70 (12.5) | 130 (18.0) | 125 (18.7) |  |
|  | 4 | 217 (8.3) | 61 (9.3) | 37 (6.6) | 57 (7.9) | 62 (9.3) |  |
| SOFA max cardio, n (%) | 0 | 1178 (36.3) | 241 (29.2) | 281 (41.4) | 497 (53.0) | 159 (19.7) | <0.001 |
|  | 1 | 607 (18.7) | 188 (22.8) | 118 (17.4) | 144 (15.4) | 157 (19.4) |  |
|  | 2 | 42 (1.3) | 14 (1.7) | 3 (0.4) | 15 (1.6) | 10 (1.2) |  |
|  | 3 | 387 (11.9) | 85 (10.3) | 111 (16.4) | 102 (10.9) | 89 (11.0) |  |
|  | 4 | 1035 (31.9) | 297 (36.0) | 165 (24.3) | 179 (19.1) | 394 (48.7) |  |
| SOFA max respiratory, n (%) | 0 | 100 (4.6) | 47 (9.6) | 8 (1.6) | 20 (3.2) | 25 (4.4) | <0.001 |
|  | 1 | 150 (7.0) | 53 (10.8) | 9 (1.9) | 44 (7.1) | 44 (7.8) |  |
|  | 2 | 307 (14.2) | 72 (14.7) | 31 (6.4) | 99 (16.0) | 105 (18.7) |  |
|  | 3 | 672 (31.2) | 158 (32.2) | 98 (20.2) | 218 (35.2) | 198 (35.2) |  |
|  | 4 | 928 (43.0) | 161 (32.8) | 339 (69.9) | 238 (38.4) | 190 (33.8) |  |

**Supplemental Table E10.** Maximum individual SOFA per phenotype in 24-hour pre-intubation region for Grady MICU data.

| **Individual SOFA Grady MICU** | **SOFA value** | **Whole cohort** | **A** | **B** | **C** | **D** | **p-value** |
| --- | --- | --- | --- | --- | --- | --- | --- |
| n | - | 867 | 214 | 49 | 404 | 200 | - |
| SOFA max coagulopathy, n (%) | 0 | 516 (61.2) | 124 (58.8) | 18 (39.1) | 278 (71.3) | 96 (49.0) | <0.001 |
|  | 1 | 145 (17.2) | 40 (19.0) | 14 (30.4) | 67 (17.2) | 24 (12.2) |  |
|  | 2 | 117 (13.9) | 23 (10.9) | 9 (19.6) | 38 (9.7) | 47 (24.0) |  |
|  | 3 | 48 (5.7) | 20 (9.5) | 4 (8.7) | 5 (1.3) | 19 (9.7) |  |
|  | 4 | 17 (2.0) | 4 (1.9) | 1 (2.2) | 2 (0.5) | 10 (5.1) |  |
| SOFA max renal, n (%) | 0 | 296 (34.4) | 21 (9.9) | 11 (22.9) | 199 (49.5) | 65 (32.8) | <0.001 |
|  | 1 | 215 (25.0) | 34 (16.0) | 14 (29.2) | 114 (28.4) | 53 (26.8) |  |
|  | 2 | 145 (16.9) | 29 (13.7) | 10 (20.8) | 61 (15.2) | 45 (22.7) |  |
|  | 3 | 66 (7.7) | 32 (15.1) | 7 (14.6) | 13 (3.2) | 14 (7.1) |  |
|  | 4 | 138 (16.0) | 96 (45.3) | 6 (12.5) | 15 (3.7) | 21 (10.6) |  |
| SOFA max hepatic, n (%) | 0 | 507 (63.9) | 147 (73.9) | 28 (62.2) | 253 (69.1) | 79 (42.9) | <0.001 |
|  | 1 | 126 (15.9) | 25 (12.6) | 3 (6.7) | 67 (18.3) | 31 (16.8) |  |
|  | 2 | 104 (13.1) | 21 (10.6) | 7 (15.6) | 38 (10.4) | 38 (20.7) |  |
|  | 3 | 33 (4.2) | 5 (2.5) | 3 (6.7) | 6 (1.6) | 19 (10.3) |  |
|  | 4 | 24 (3.0) | 1 (0.5) | 4 (8.9) | 2 (0.5) | 17 (9.2) |  |
| SOFA max neuro, n (%) | 0 | 255 (32.2) | 53 (26.9) | 19 (42.2) | 112 (30.9) | 71 (37.8) | 0.017 |
|  | 1 | 138 (17.4) | 31 (15.7) | 14 (31.1) | 57 (15.7) | 36 (19.1) |  |
|  | 2 | 136 (17.2) | 38 (19.3) | 7 (15.6) | 64 (17.6) | 27 (14.4) |  |
|  | 3 | 186 (23.5) | 50 (25.4) | 4 (8.9) | 90 (24.8) | 42 (22.3) |  |
|  | 4 | 78 (9.8) | 25 (12.7) | 1 (2.2) | 40 (11.0) | 12 (6.4) |  |
| SOFA max cardio, n (%) | 0 | 449 (51.8) | 71 (33.2) | 19 (38.8) | 296 (73.4) | 63 (31.5) | <0.001 |
|  | 1 | 190 (21.9) | 61 (28.5) | 14 (28.6) | 62 (15.4) | 53 (26.5) |  |
|  | 2 | 7 (0.8) | 4 (1.9) | 0 | 1 (0.2) | 2 (1.0) |  |
|  | 3 | 22 (2.5) | 8 (3.7) | 2 (4.1) | 6 (1.5) | 6 (3.0) |  |
|  | 4 | 198 (22.9) | 70 (32.7) | 14 (28.6) | 38 (9.4) | 76 (38.0) |  |
| SOFA max respiratory, n (%) | 0 | 27 (5.4) | 5 (4.2) | 0 | 14 (6.4) | 8 (6.5) | 0.009 |
|  | 1 | 38 (7.7) | 16 (13.3) | 0 | 14 (6.4) | 8 (6.5) |  |
|  | 2 | 89 (17.9) | 30 (25.0) | 4 (12.5) | 34 (15.5) | 21 (16.9) |  |
|  | 3 | 174 (35.1) | 43 (35.8) | 11 (34.4) | 82 (37.3) | 38 (30.6) |  |
|  | 4 | 168 (33.9) | 26 (21.7) | 17 (53.1) | 76 (34.5) | 49 (39.5) |  |

**Supplemental Table E11.** Maximum individual SOFA per phenotype in 24-hour pre-intubation region for Emory SICU data.

| **Individual SOFA Emory SICU** | **SOFA value** | **Whole cohort** | **A** | **B** | **C** | **D** | **p-value** |
| --- | --- | --- | --- | --- | --- | --- | --- |
| n | - | 1128 | 818 | 39 | 259 | 12 | - |
| SOFA max coagulopathy, n (%) | 0 | 633 (58.9) | 432 (55.7) | 24 (64.9) | 173 (69.2) | 4 (33.3) | 0.006 |
|  | 1 | 196 (18.2) | 152 (19.6) | 8 (21.6) | 34 (13.6) | 2 (16.7) |  |
|  | 2 | 156 (14.5) | 123 (15.9) | 3 (8.1) | 28 (11.2) | 2 (16.7) |  |
|  | 3 | 71 (6.6) | 55 (7.1) | 1 (2.7) | 12 (4.8) | 3 (25.0) |  |
|  | 4 | 18 (1.7) | 13 (1.7) | 1 (2.7) | 3 (1.2) | 1 (8.3) |  |
| SOFA max renal, n (%) | 0 | 453 (42.1) | 268 (34.4) | 17 (47.2) | 162 (64.8) | 6 (54.5) | <0.001 |
|  | 1 | 282 (26.2) | 202 (25.9) | 12 (33.3) | 67 (26.8) | 1 (9.1) |  |
|  | 2 | 163 (15.1) | 140 (18.0) | 3 (8.3) | 18 (7.2) | 2 (18.2) |  |
|  | 3 | 65 (6.0) | 59 (7.6) | 2 (5.6) | 2 (0.8) | 2 (18.2) |  |
|  | 4 | 113 (10.5) | 110 (14.1) | 2 (5.6) | 1 (0.4) | 0 |  |
| SOFA max hepatic, n (%) | 0 | 546 (64.0) | 372 (62.4) | 20 (64.5) | 151 (69.9) | 3 (30.0) | 0.001 |
|  | 1 | 138 (16.2) | 90 (15.1) | 6 (19.4) | 39 (18.1) | 3 (30.0) |  |
|  | 2 | 114 (13.4) | 86 (14.4) | 3 (9.7) | 24 (11.1) | 1 (10.0) |  |
|  | 3 | 31 (3.6) | 26 (4.4) | 2 (6.5) | 2 (0.9) | 1 (10.0) |  |
|  | 4 | 24 (2.8) | 22 (3.7) | 0 | 0 | 2 (20.0) |  |
| SOFA max neuro, n (%) | 0 | 307 (38.0) | 221 (37.1) | 14 (43.8) | 69 (40.4) | 3 (33.3) | 0.902 |
|  | 1 | 179 (22.2) | 135 (22.7) | 8 (25.0) | 34 (19.9) | 2 (22.2) |  |
|  | 2 | 129 (16.0) | 99 (16.6) | 3 (9.4) | 25 (14.6) | 2 (22.2) |  |
|  | 3 | 95 (11.8) | 67 (11.2) | 5 (15.6) | 21 (12.3) | 2 (22.2) |  |
|  | 4 | 98 (12.1) | 74 (12.4) | 2 (6.2) | 22 (12.9) | 0 |  |
| SOFA max cardio, n (%) | 0 | 312 (28.2) | 199 (24.5) | 16 (42.1) | 96 (38.9) | 1 (9.1) | 0.001 |
|  | 1 | 161 (14.5) | 117 (14.4) | 9 (23.7) | 34 (13.8) | 1 (9.1) |  |
|  | 2 | 8 (0.7) | 7 (0.9) | 0 | 1 (0.4) | 0 |  |
|  | 3 | 108 (9.8) | 83 (10.2) | 4 (10.5) | 20 (8.1) | 1 (9.1) |  |
|  | 4 | 518 (46.8) | 405 (49.9) | 9 (23.7) | 96 (38.9) | 8 (72.7) |  |
| SOFA max respiratory, n (%) | 0 | 71 (8.4) | 65 (10.9) | 0 | 6 (3.0) | 0 | <0.001 |
|  | 1 | 81 (9.6) | 67 (11.2) | 0 | 14 (6.9) | 0 |  |
|  | 2 | 147 (17.4) | 112 (18.7) | 0 | 33 (16.3) | 2 (20.0) |  |
|  | 3 | 258 (30.5) | 180 (30.1) | 3 (8.6) | 71 (35.0) | 4 (40.0) |  |
|  | 4 | 289 (34.2) | 174 (29.1) | 32 (91.4) | 79 (38.9) | 4 (40.0) |  |

**Supplemental Table E12.** Maximum individual SOFA per phenotype in 24-hour pre-intubation region for Grady SICU data.

| **Individual SOFA Grady SICU** | **SOFA value** | **Whole cohort** | **A** | **B** | **C** | **D** | **p-value** |
| --- | --- | --- | --- | --- | --- | --- | --- |
| n | - | 466 | 133 | 5 | 264 | 64 | - |
| SOFA max coagulopathy, n (%) | 0 | 306 (68.5) | 86 (67.7) | 4 (80.0) | 188 (74.0) | 28 (45.9) | <0.001 |
|  | 1 | 75 (16.8) | 23 (18.1) | 0 | 38 (15.0) | 14 (23.0) |  |
|  | 2 | 49 (11.0) | 15 (11.8) | 1 (20.0) | 21 (8.3) | 12 (19.7) |  |
|  | 3 | 14 (3.1) | 3 (2.4) | 0 | 7 (2.8) | 4 (6.6) |  |
|  | 4 | 3 (0.7) | 0 | 0 | 0 | 3 (4.9) |  |
| SOFA max renal, n (%) | 0 | 176 (38.7) | 14 (10.9) | 2 (40.0) | 137 (52.9) | 23 (37.1) | <0.001 |
|  | 1 | 135 (29.7) | 26 (20.2) | 2 (40.0) | 85 (32.8) | 22 (35.5) |  |
|  | 2 | 81 (17.8) | 38 (29.5) | 0 | 32 (12.4) | 11 (17.7) |  |
|  | 3 | 21 (4.6) | 15 (11.6) | 0 | 2 (0.8) | 4 (6.5) |  |
|  | 4 | 42 (9.2) | 36 (27.9) | 1 (20.0) | 3 (1.2) | 2 (3.2) |  |
| SOFA max hepatic, n (%) | 0 | 262 (66.5) | 81 (72.3) | 3 (60.0) | 151 (68.9) | 27 (46.6) | 0.006 |
|  | 1 | 59 (15.0) | 13 (11.6) | 1 (20.0) | 35 (16.0) | 10 (17.2) |  |
|  | 2 | 65 (16.5) | 18 (16.1) | 1 (20.0) | 30 (13.7) | 16 (27.6) |  |
|  | 3 | 7 (1.8) | 0 | 0 | 3 (1.4) | 4 (6.9) |  |
|  | 4 | 1 (0.3) | 0 | 0 | 0 | 1 (1.7) |  |
| SOFA max neuro, n (%) | 0 | 157 (37.0) | 43 (35.8) | 2 (40.0) | 87 (35.8) | 25 (44.6) | 0.921 |
|  | 1 | 64 (15.1) | 21 (17.5) | 1 (20.0) | 32 (13.2) | 10 (17.9) |  |
|  | 2 | 80 (18.9) | 19 (15.8) | 1 (20.0) | 50 (20.6) | 10 (17.9) |  |
|  | 3 | 88 (20.8) | 26 (21.7) | 1 (20.0) | 53 (21.8) | 8 (14.3) |  |
|  | 4 | 35 (8.3) | 11 (9.2) | 0 | 21 (8.6) | 3 (5.4) |  |
| SOFA max cardio, n (%) | 0 | 253 (54.8) | 48 (36.4) | 1 (20.0) | 194 (74.3) | 10 (15.6) | <0.001 |
|  | 1 | 110 (23.8) | 42 (31.8) | 2 (40.0) | 34 (13.0) | 32 (50.0) |  |
|  | 3 | 19 (4.1) | 5 (3.8) | 0 | 9 (3.4) | 5 (7.8) |  |
|  | 4 | 80 (17.3) | 37 (28.0) | 2 (40.0) | 24 (9.2) | 17 (26.6) |  |
| SOFA max respiratory, n (%) | 0 | 25 (8.5) | 13 (15.3) | 0 | 9 (5.6) | 3 (6.4) | 0.12 |
|  | 1 | 47 (15.9) | 18 (21.2) | 0 | 22 (13.8) | 7 (14.9) |  |
|  | 2 | 70 (23.7) | 21 (24.7) | 0 | 40 (25.0) | 9 (19.1) |  |
|  | 3 | 73 (24.7) | 17 (20.0) | 1 (33.3) | 39 (24.4) | 16 (34.0) |  |
|  | 4 | 80 (27.1) | 16 (18.8) | 2 (66.7) | 50 (31.2) | 12 (25.5) |  |

**Supplemental Table E13.** List of confounders used for propensity score matching (PSM).

| **Routine labs, vital signs, demographics and clinical scores: variable names and their meanings** | |
| --- | --- |
| c1 - temperature: Body temperature (^o^C)  c2 - sbp_cuff: Cuff-based systolic blood pressure (mmHg)  c3 - dbp_cuff: Cuff-based diastolic blood pressure (mmHg)  c4 - pulse: Pulse rate (beats per minute)  c5 - unassisted_resp_rate: Respiratory rate (breaths/min.)  c6 - spo2: Blood saturated oxygen concentration, SpO_2_ level (%)  c7 - end_tidal_co2: End-tidal CO_2_ (mmHg)  c8 - o2_flow_rate: (L/minute)  c9 - bicarb_(hco3): (mmol/L)  c10 - blood_urea_nitrogen_(bun): (mg/dL)  c11 - calcium (mg/dL)  c12 - calcium_ionized (mg/dL)  c13 - chloride (mEq/L)  c14 - creatinine: (mg/dL)  c15 - glucose (mmol/L)  c16 - magnesium (mg/dL)  c17 - osmolarity (mOsm/kg)  c18 - phosphorus (mg/dL)  c19 - potassium (mEq/L)  c20 - sodium (mEq/L)  c21 - hemoglobin: (g/dL)  c22 - met_hgb: (g/dL)  c23 - platelets: (×10^9^/L)  c24 - white_blood_cell_count: (×10^9^/L)  c25 - carboxy_hgb (%)  c26 - alanine_aminotransferase_(alt) (U/L) | c27 - albumin: (g/L)  c28 - alkaline_phosphatase (IU/L)  c29 - ammonia: (µg/dL)  c30 - bilirubin_direct: (mg/dL)  c31 - bilirubin_total: (mg/dL)  c32 - fibrinogen: (mg/dL)  c33 - inr: International Normalized Ratio  c34 - lactate_dehydrogenase: (IU/L)  c35 - lactic_acid: (mmol/L)  c36 - partial_prothrombin_time_(ptt): (s)  c37 - protein: (g/dL)  c38 - lipase: (U/L)  c39 - b-type_natriuretic_peptide_(bnp): B-type natriuretic peptide (pg/ml)  c40 - troponin: (ng/mL)  c41 - fio2: Fraction of inspired oxygen (range: 0-1)  c42 - partial_pressure_of_carbon_dioxide_(paco2):  PaCO_2_ (mmHg)  c43 - partial_pressure_of_oxygen_(pao2): PaO_2_ (mmHg)  c44 - ph  c45 - saturation_of_oxygen_(sao2) (%)  c46 - d_dimer: (ng/mL)  c47 - hemoglobin_a1c (%)  c48 - best_map: Mean arterial pressure (mmHg)  c49 - pf_sp: SpO2/FiO2 ratio  c50 - pf_pa: PaO2/FiO2 ratio (mmHg)  c51 - age: Age (years)  c52-54 - Race variables (White / Black / Others) (0/1)  c55 - Sex_Male: (0/1)  c56 - SOFA_max_hourly_total: Maximum total SOFA  c57 - gcs_total_score: Total GCS score |

**Supplemental Table E14.** Characteristics of treatment analysis for high PEEP regime**.**

| **Characteristics** | **Description** |
| --- | --- |
| Propensity score model | Logistic regression with liblinear solver |
| Matching type | 1:1 for treated and controls, drop unmatched |
| Caliper radius for matching | 0.4 × standard deviation of propensity logits |
| Treatment variable | High PEEP: 1, Low PEEP: 0 |
| Outcome variable | 28-day short-term mortality |
| Analysis methods | ATE, effect size, Kaplan Meier analysis |


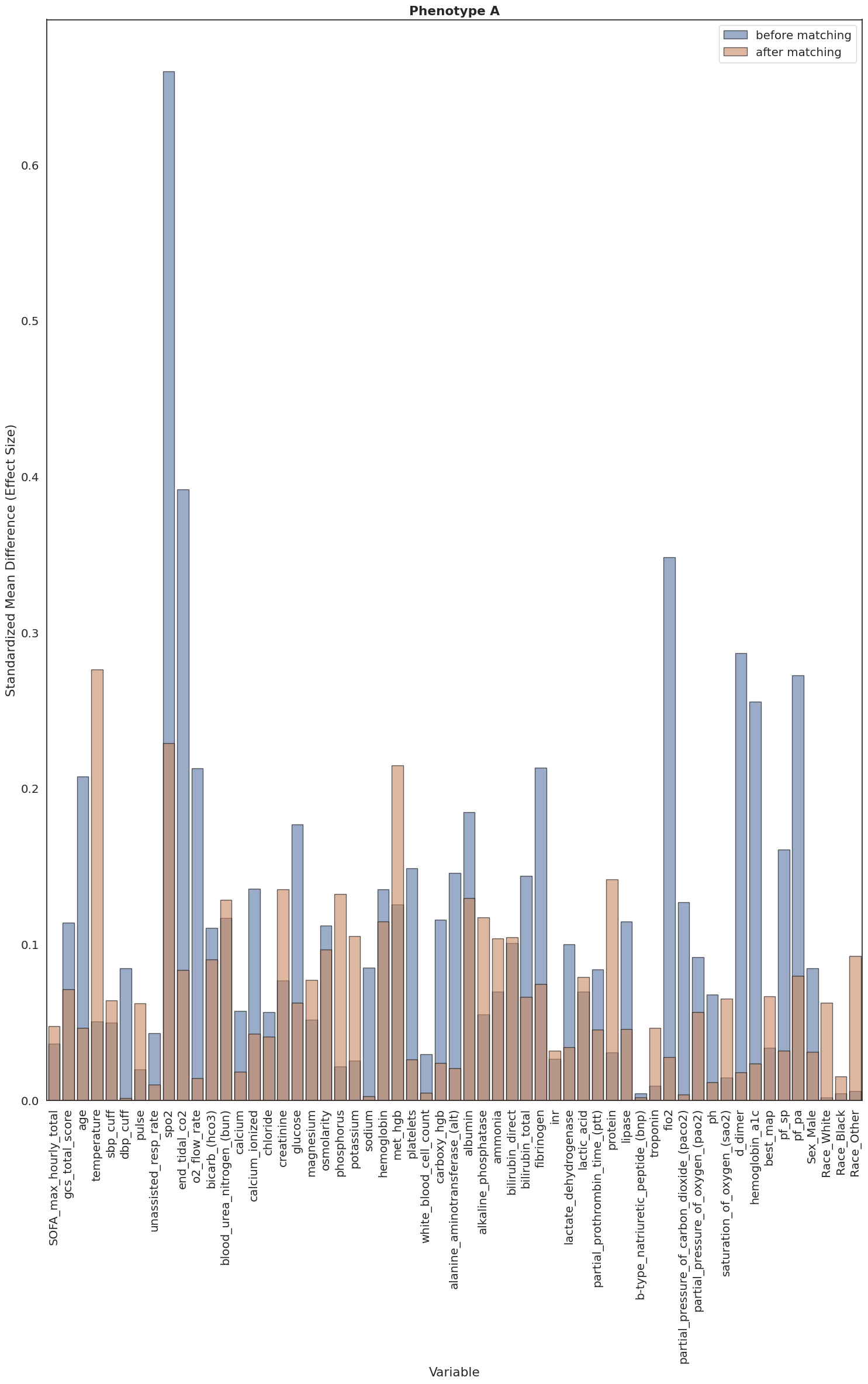


**Supplemental Figure E8.** Effect size for phenotype A of the derivation set.


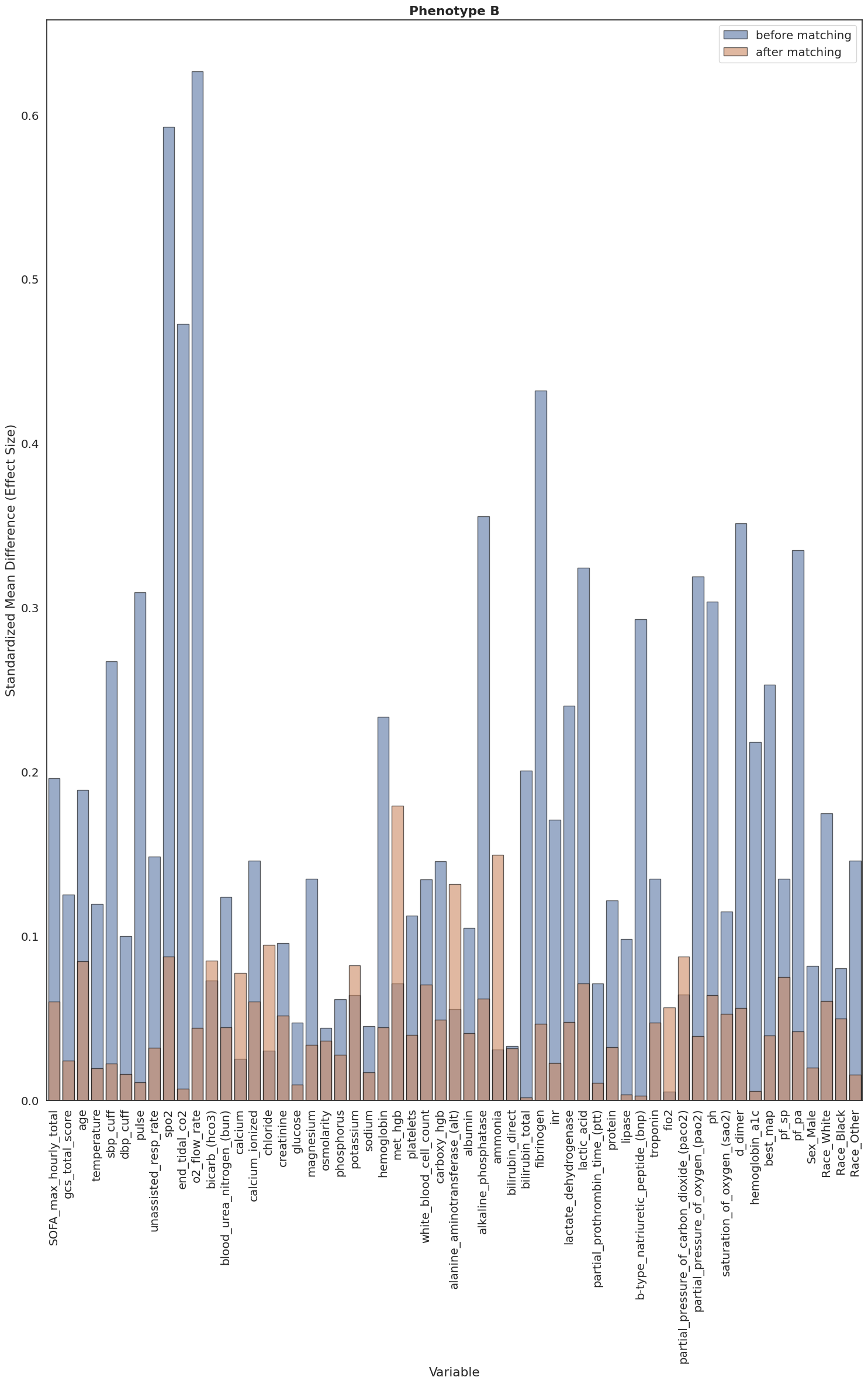


**Supplemental Figure E9.** Effect size for phenotype B of the derivation set.


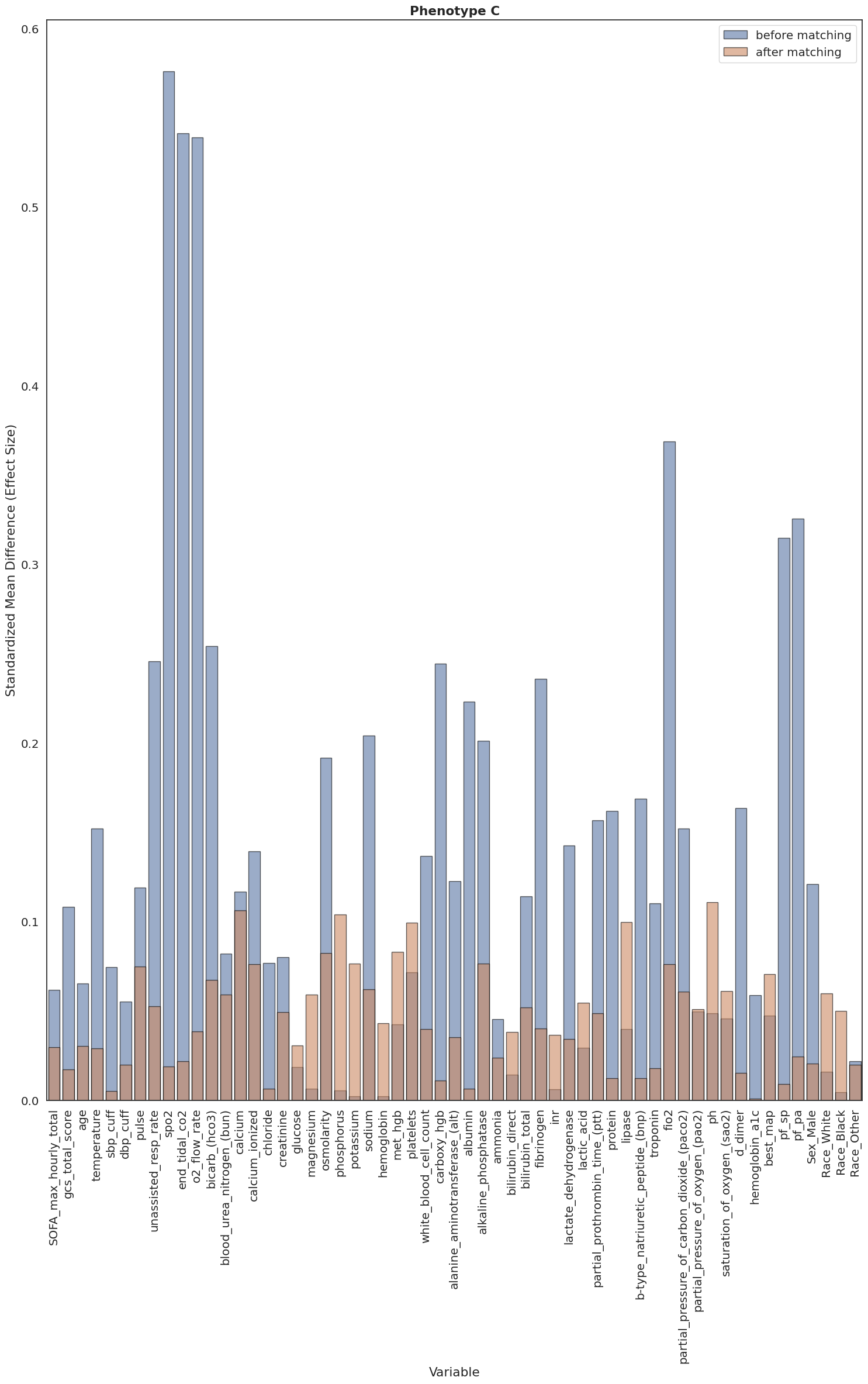


**Supplemental Figure E10.** Effect size for phenotype C of the derivation set.


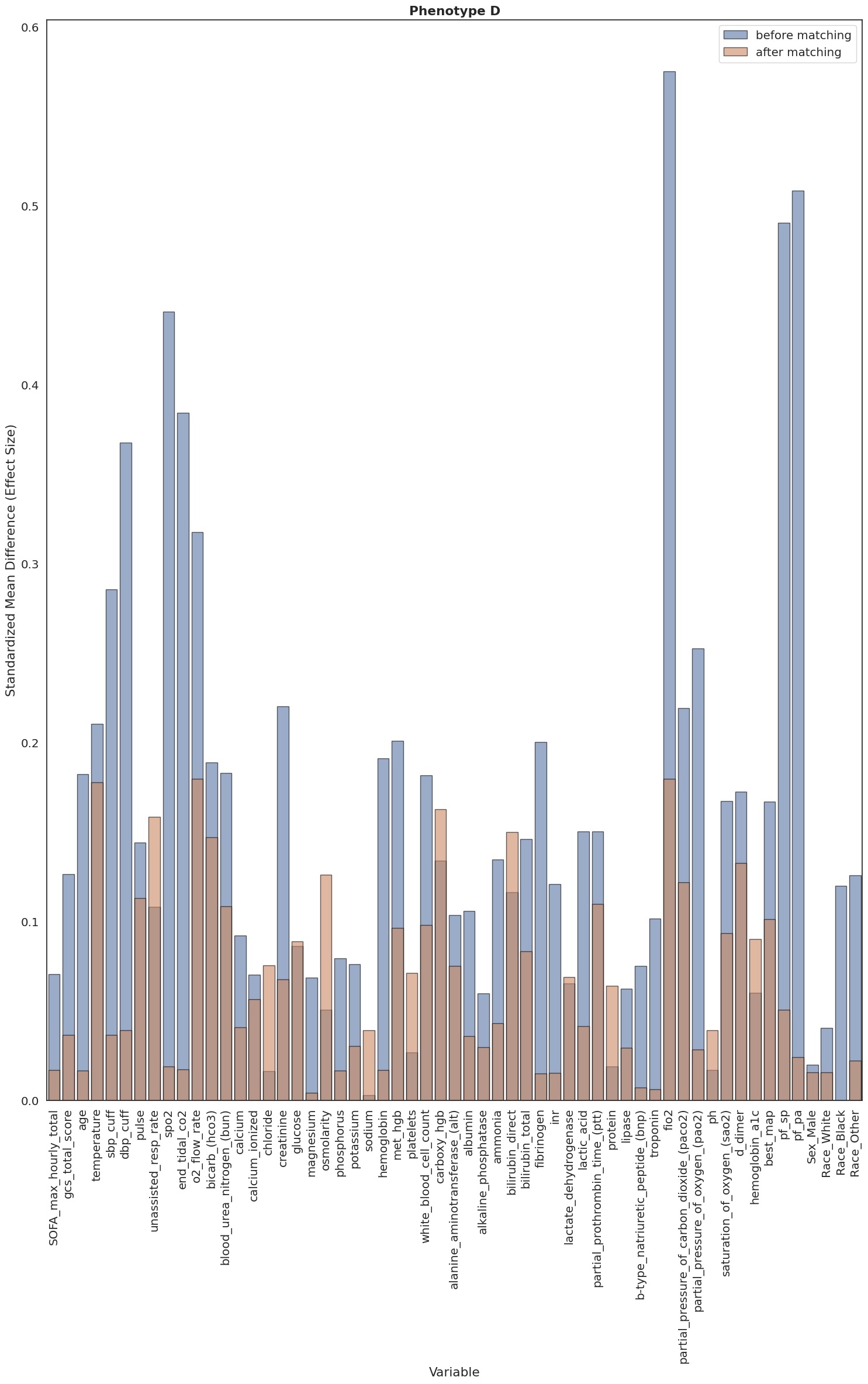


**Supplemental Figure E11.** Effect size for phenotype D of the derivation set.
